# Light adaptation controls visual sensitivity by adjusting the speed and gain of the response to light

**DOI:** 10.1371/journal.pone.0220358

**Published:** 2019-08-07

**Authors:** Andrew T. Rider, G. Bruce Henning, Andrew Stockman

**Affiliations:** UCL Institute of Ophthalmology, University College London, London, England; Doheny Eye Institute/UCLA, UNITED STATES

## Abstract

The range of c. 10^12^ ambient light levels to which we can be exposed massively exceeds the <10^3^ response range of neurons in the visual system, but we can see well in dim starlight and bright sunlight. This remarkable ability is achieved largely by a speeding up of the visual response as light levels increase, causing characteristic changes in our sensitivity to different rates of flicker. Here, we account for over 65 years of flicker-sensitivity measurements with an elegantly-simple, physiologically-relevant model built from first-order low-pass filters and subtractive inhibition. There are only two intensity-dependent model parameters: one adjusts the speed of the visual response by shortening the time constants of some of the filters in the direct cascade as well as those in the inhibitory stages; the other parameter adjusts the overall gain at higher light levels. After reviewing the physiological literature, we associate the variable gain and three of the variable-speed filters with biochemical processes in cone photoreceptors, and a further variable-speed filter with processes in ganglion cells. The variable-speed but fixed-strength subtractive inhibition is most likely associated with lateral connections in the retina. Additional fixed-speed filters may be more central. The model can explain the important characteristics of human flicker-sensitivity including the approximate dependences of low-frequency sensitivity on contrast (Weber’s law) and of high-frequency sensitivity on amplitude (“high-frequency linearity”), the exponential loss of high-frequency sensitivity with increasing frequency, and the logarithmic increase in temporal acuity with light level (Ferry-Porter law). In the time-domain, the model can account for several characteristics of flash sensitivity including changes in contrast sensitivity with light level (de Vries-Rose and Weber’s laws) and changes in temporal summation (Bloch’s law). The new model provides fundamental insights into the workings of the visual system and gives a simple account of many visual phenomena.

## Introduction

A primary goal of visual sensitivity-regulation or light adaptation is to enable the visual system to perform effectively over light levels that can vary by more than 10^12^ despite the dynamic ranges of neurons in the visual pathway being limited to 10^3^ or less (e.g. [1, 2]). This biological challenge is, of course, made more manageable by partitioning the task into the overlapping ranges served by the sluggish, highly sensitive rod system and by the faster, less sensitive cone system [3–6]. Nevertheless, the cone system must still operate over a range of >10^8^ (e.g., Table 5.1 in [7]). In order to prevent later postreceptoral neurons from exceeding their limited dynamic range and saturating, light adaptation at moderate and high light levels must occur primarily at or before the synapses in the cone pedicles.

Our ability to see well over such a wide range of light levels is achieved primarily by the speeding up of the visual response as the mean light level increases [8–13]. As will be seen, this can be achieved by adjustments in the speed of a relatively small number of simple biochemical processes. The perceptual effects of such speed adjustments produce characteristic changes in our sensitivity to flickering light with changes in light level. Here, we bring together 65 years of flicker-sensitivity measurements to provide a new model of light adaptation with just two level-dependent parameters. One parameter of the model controls speed, and the other controls gain.

**Fig 1:**
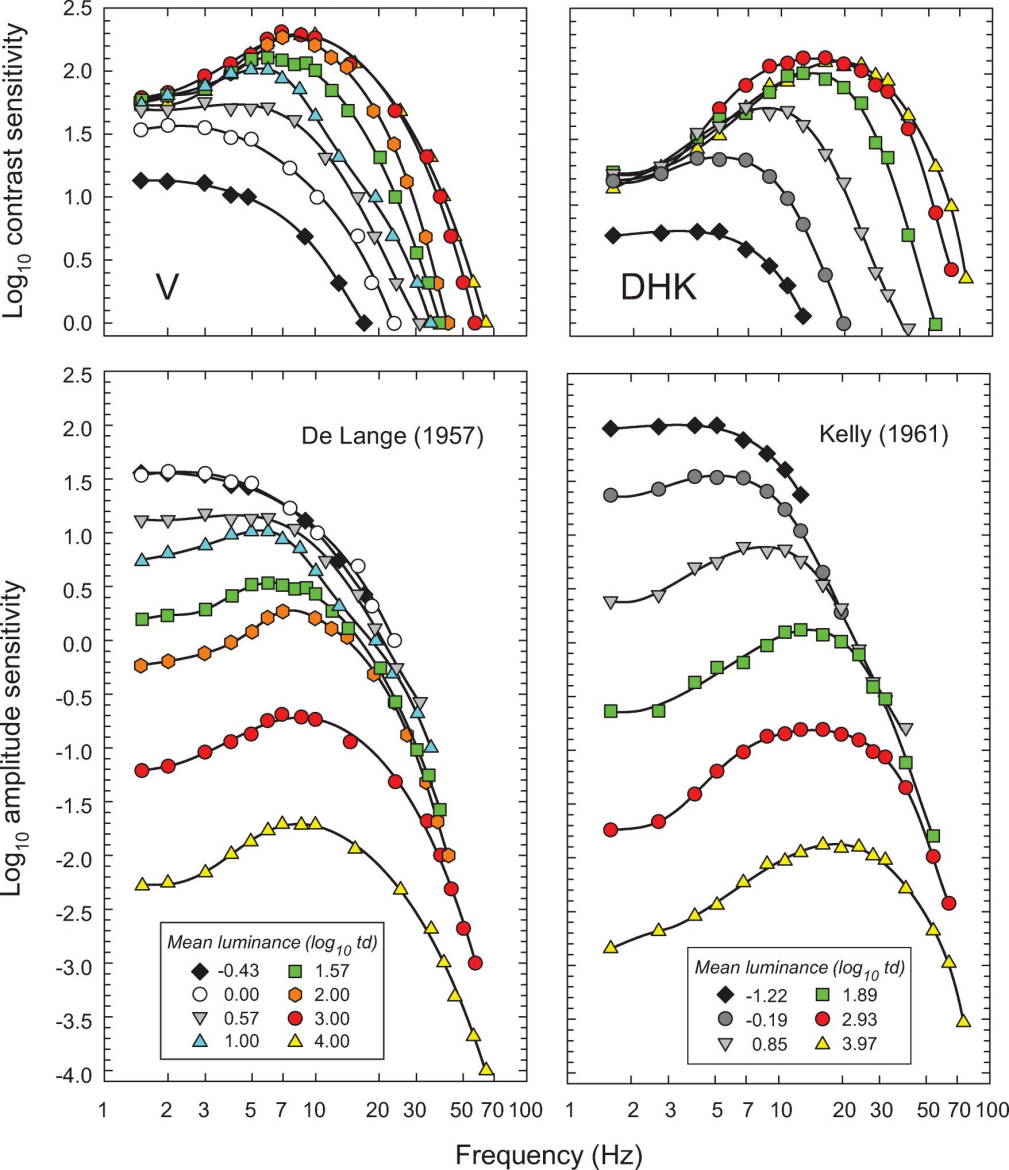
The left-hand column shows data for observer V from De Lange [14] and the right-hand column, data for observer DHK from Kelly [15] both measured using sinusoidally flickering stimuli. The data are shown twice: The upper panels show the logarithm of the reciprocal of the just-detectable contrast (the contrast sensitivity) as a function of frequency (Hz) plotted on a logarithmic scale. The lower panels show the same data replotted as the logarithm of the reciprocal of the just-detectable amplitude (the amplitude sensitivity) also as a function of frequency (logarithmic scale). Different symbols and colors denote different mean retinal illuminances (log_10_ photopic trolands) as indicated in the key. The black curves are arbitrary smooth functions fitted to each dataset to facilitate comparison. Data are from Figure 5 in De Lange [14], who used a 2° diameter, centrally fixated, white flickering test field in a steady 60° diameter surround of same luminance and chromaticity, and from data tabulated in Table 1 in Kelly [15], who used a centrally-fixated, 50° diameter white target vignetted gradually from 50 to 68°.

Fig 1 shows two often reproduced classic sets of “temporal contrast-sensitivity functions” (TCSFs). Each curve shows, for a single observer, how sensitivity to sinusoidal flicker depends on temporal frequency; the different curves are for different mean light levels. Each curve was obtained by presenting an observer with sinusoidally flickering stimuli of fixed mean intensity at several different flicker frequencies. At each frequency, the observer adjusted the flicker contrast to find the smallest contrast at which the flicker could just be seen—the “threshold” contrast. (Contrast, for sinusoidal flicker that extends equally above and below its mean intensity, is simply the amplitude of the flicker divided by the mean level around which the light varies. Consequently "contrast sensitivity", the reciprocal of the threshold contrast, increases upwards in the figures.)

The upper panels of Fig 1 show the logarithm of contrast sensitivity for a series of mean light levels. The thresholds in the left panels were set by De Lange’s observer V in 1958, and in the right panels by Kelly’s observer DHK in 1961 [14, 15]; the key in the lower panels gives the mean levels in log_10_ trolands (a measure of retinal illumination). Two of the principal light-dependent changes in temporal sensitivity are evident in the upper panels. At higher frequencies there are increases in the contrast sensitivity to high-frequency flicker as the mean level increases, which are consistent with the visual response speeding up and so enabling observers to see flicker at higher and higher frequencies. On the other hand, at lower frequencies contrast sensitivity is roughly independent of light level, except at the very lowest light levels. As a result, near-threshold, the contrast of steadily illuminated objects whose images are stationary on the retina should appear roughly independent of the mean light level. This independence is a manifestation of Weber’s Law [16]. As we discuss below, the obedience to Weber’s Law for several other observers under these conditions is only approximate.

**Fig 2:**
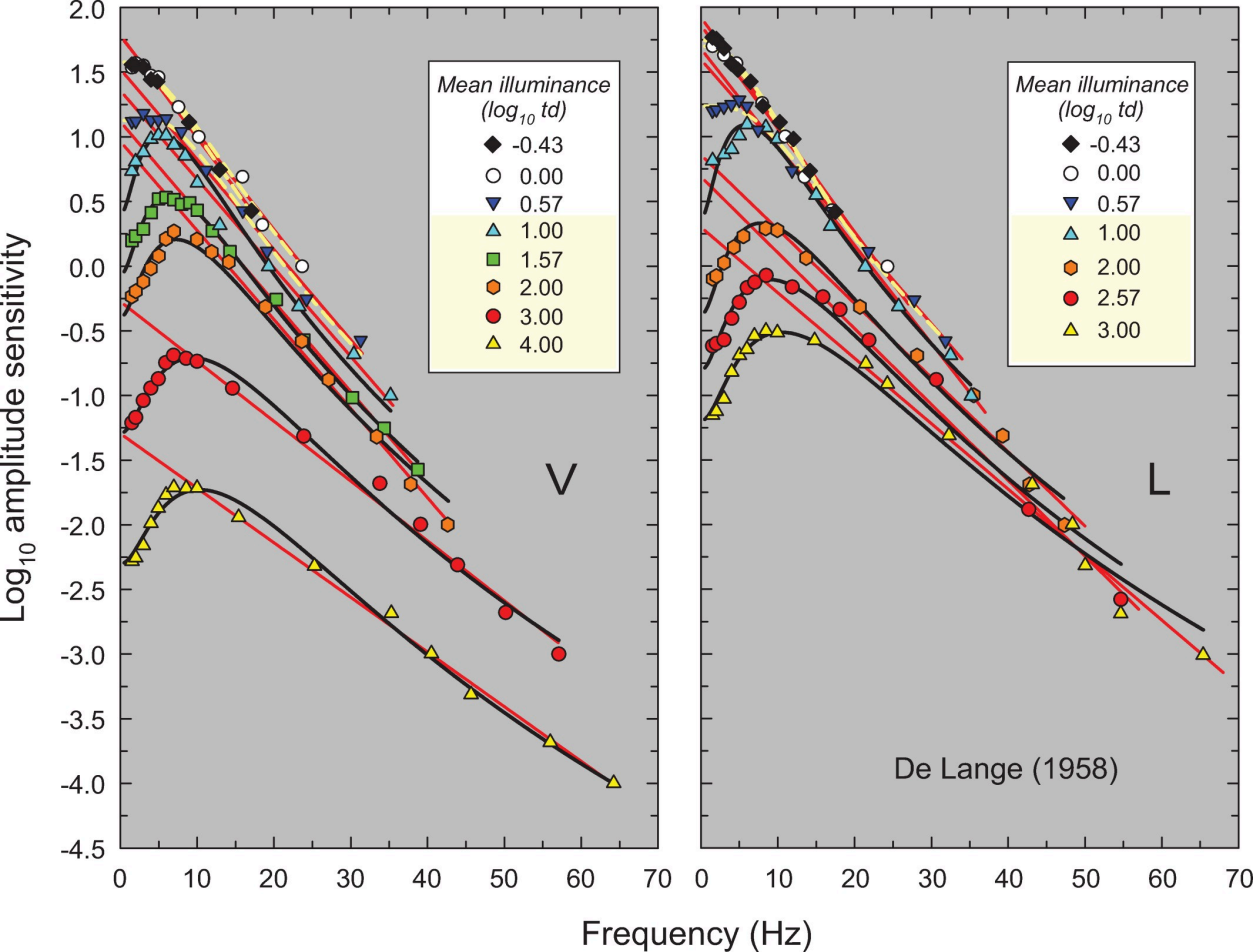
Log_10_ amplitude sensitivities for observers V (left panel) and L (right panel) measured at the eight (V) or seven (L) mean retinal illuminances (log_10_ photopic trolands) noted in the key. The sensitivities are plotted as a function of frequency (Hz), which in this figure and Figs 3–6 is shown on a linear frequency scale. Data replotted from Figures 5 and 6 in De Lange [14]. De Lange used a 2° diameter, centrally fixated, white flickering test field in a steady 60° diameter surround of same luminance and chromaticity. The solid red lines are best least-squares linear fits to the high-frequency region of each curve. The solid black and dashed yellow lines show fits of our light adaptation model. The dashed yellow lines indicate those (mesopic) levels for which the flicker detection is likely to be mediated by rods and cones, while the black lines indicate those (photopic) levels for which the detection is likely to be mediated solely by cones. (The levels thought to be photopic are also highlighted in yellow in the key).

**Fig 3:**
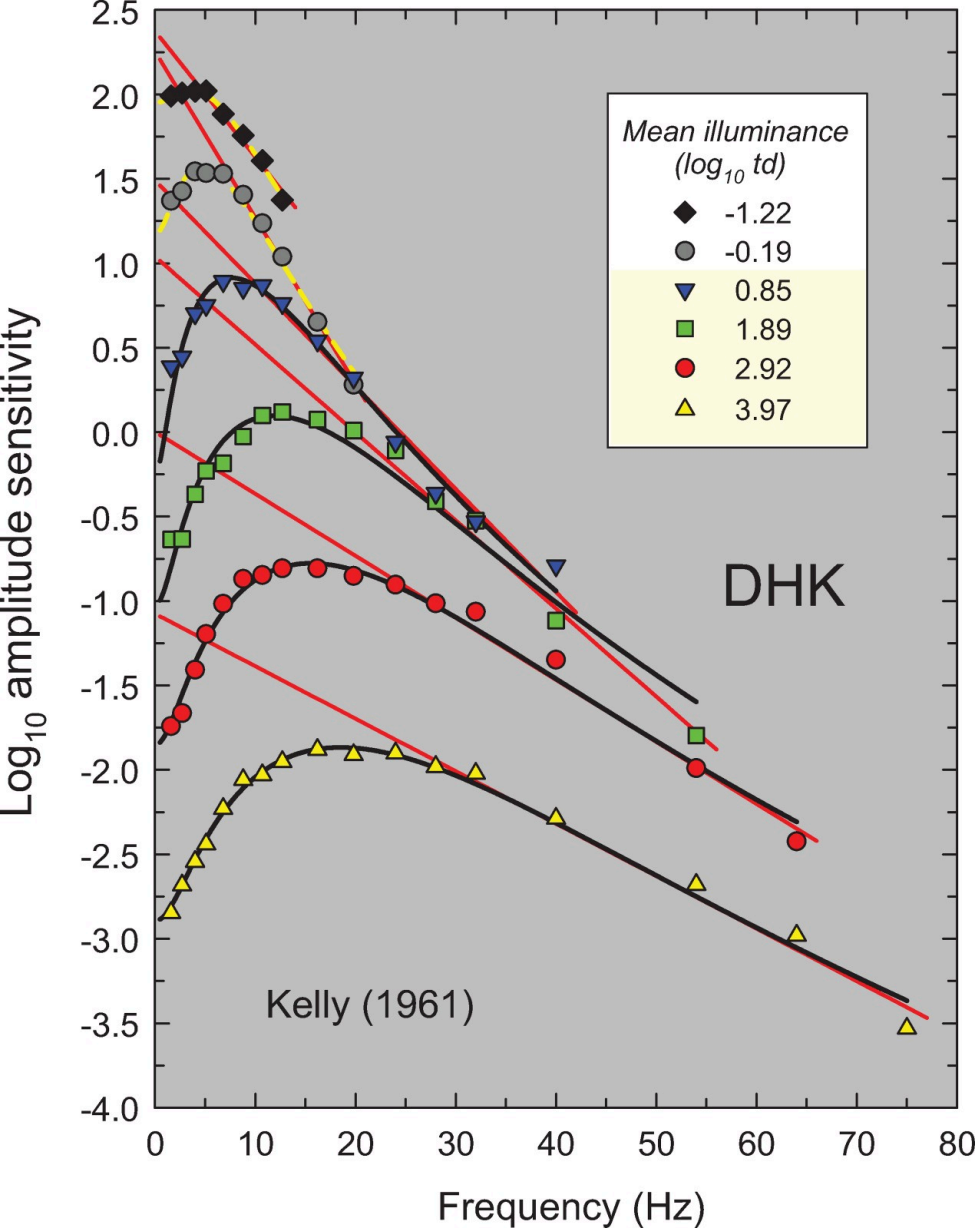
Same as Fig 2, but amplitude sensitivities for observer DHK from Table 1 in Kelly [15]. Kelly used a 50° diameter white target vignetted from 50 to 68°.

**Fig 4:**
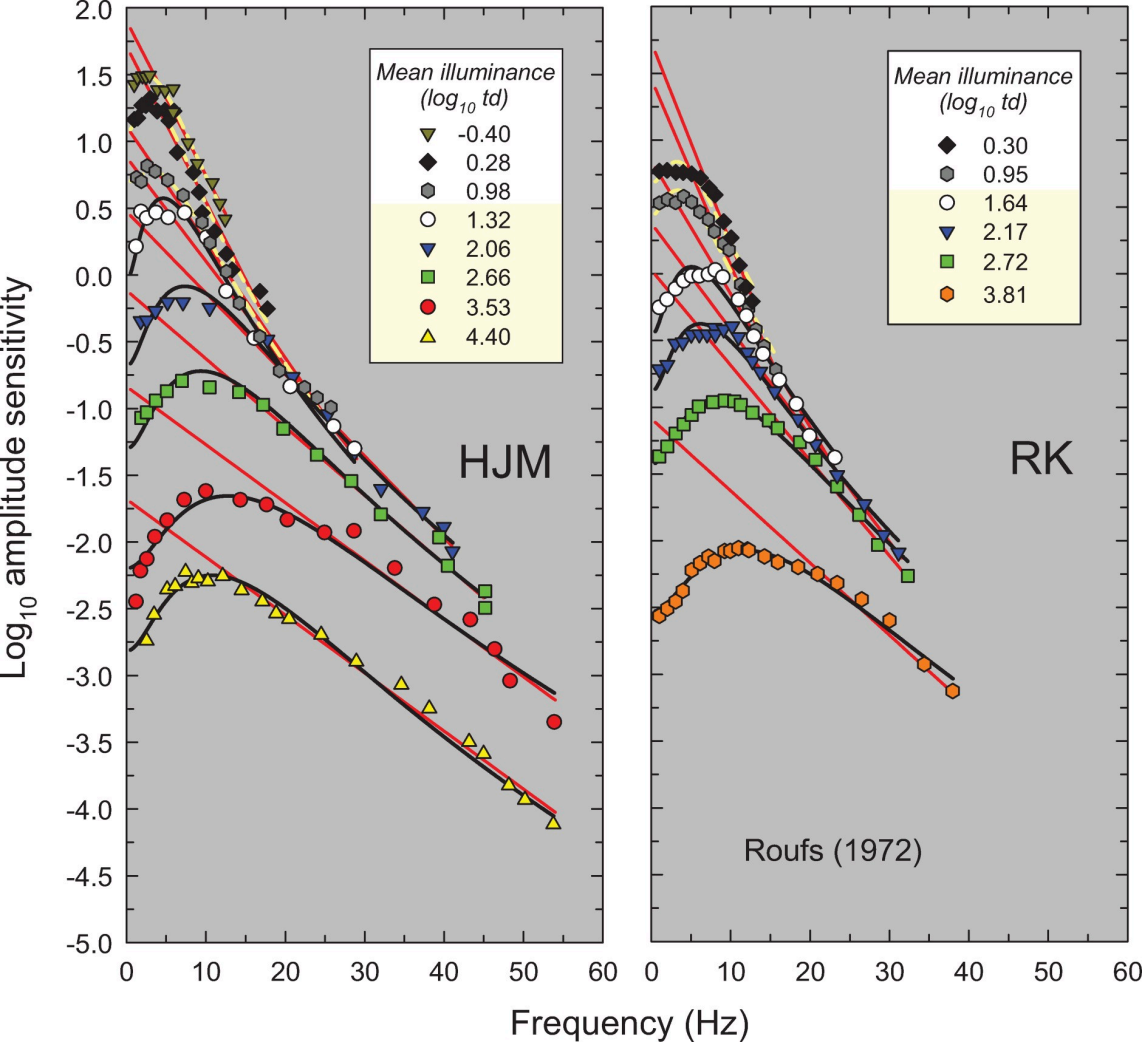
Same as Fig 2, but amplitudes sensitivities for observers HJM (left panel) and RK (right panel) from Figure 3 in Roufs [17]. Roufs used centrally fixated, 1° diameter “practically” white target with no surround.

**Fig 5:**
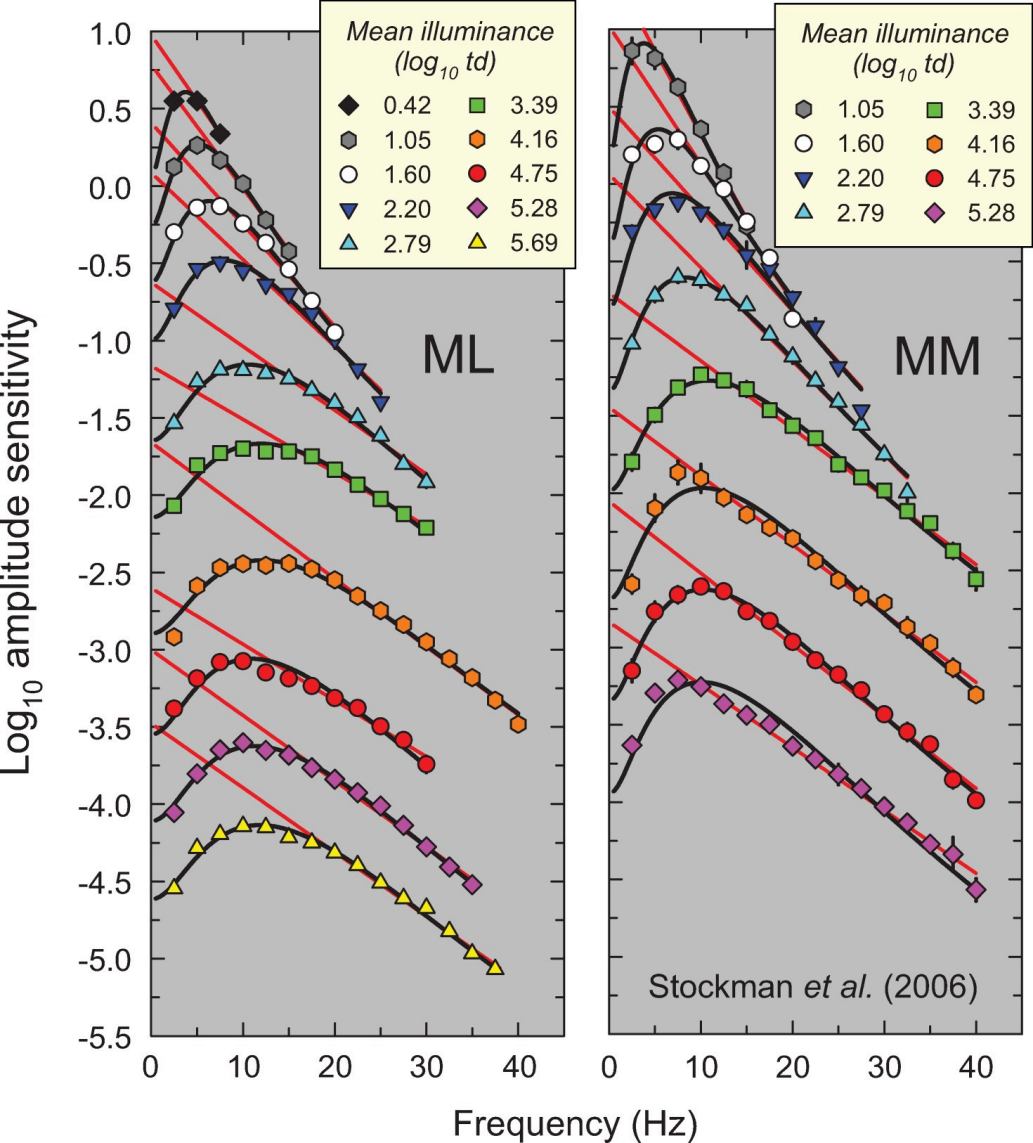
Same as Fig 2, but amplitude sensitivities for protanopic observers ML (left panel) and MM (right panel) provided by AS, originally shown in Figures 3 and 4 in Stockman *et al*. [13]. Stockman *et al*. used a centrally-fixated, 4° diameter, 610-nm target superimposed on 9° diameter 540-nm background. The ratio of the target and background radiances was fixed to produce a maximum M-cone contrast of 13%. The error bars are ±1 standard error of the mean. This combination of background and target radiances helped to eliminate rod intrusion at low retinal illuminances (as confirmed in their bleaching control experiments).

**Fig 6:**
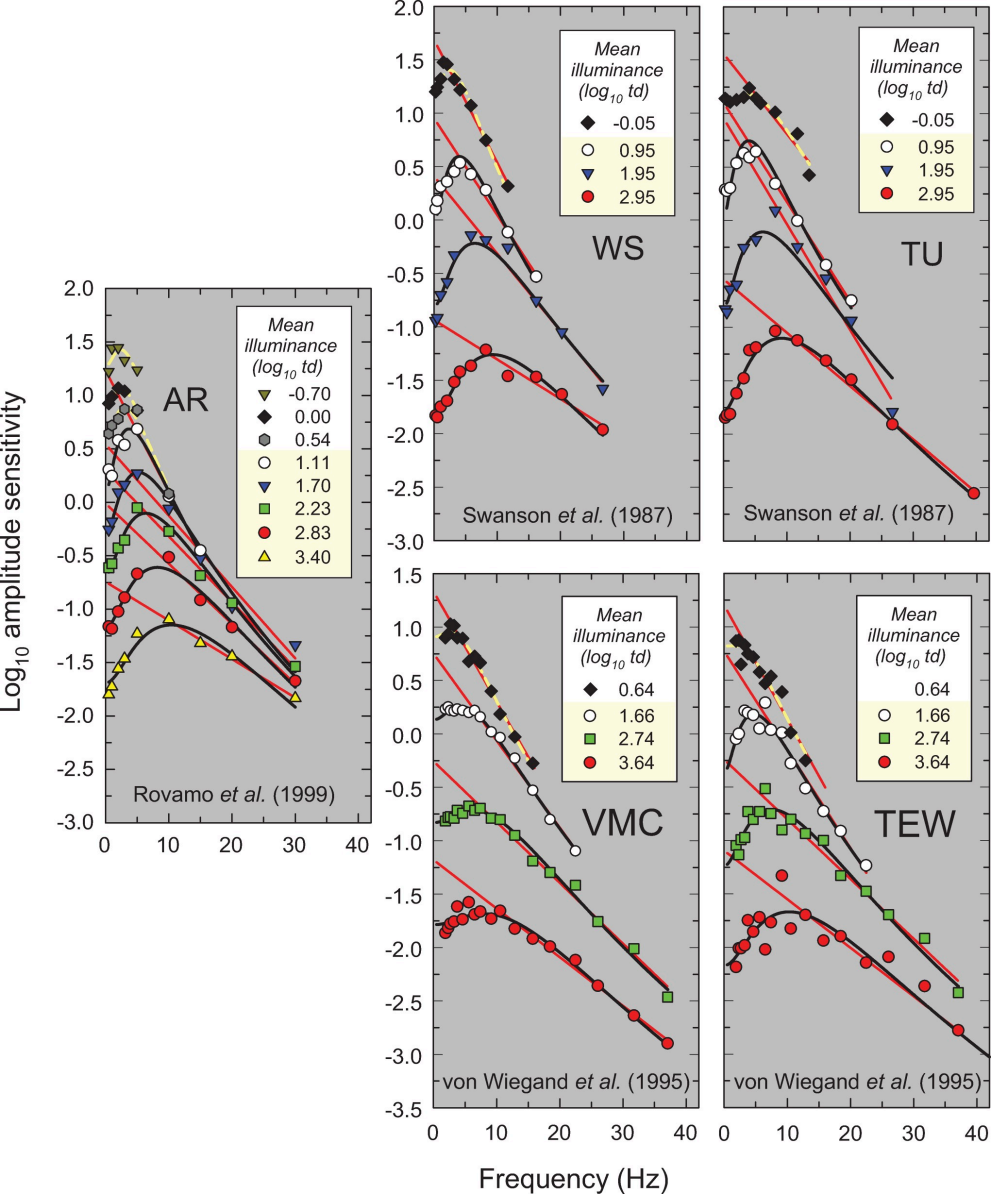
Same as Fig 2 for data from three sources. First, observer AR replotted from Figure 2 of Rovamo *et al*. [18] measured using a white 1.66° diameter flickering target inside a 3.32° diameter equiluminant white surround. Second, observers WS and TU replotted from Figure 2 in Swanson *et al*. [19] measured using a 2° diameter flickering target illuminated by a mixture of red and green LEDs (light-emitting-diodes) that appeared metameric with a 600-nm light. Third, observers VMC and TEW replotted from Figure 2 in von Wiegand *et al*. [20] measured using a flickering LED target with a dominant wavelength of 625-nm that extended to 1° diameter and then fell with a cosine intensity profile to zero intensity by 2° centred within a larger 18° diameter annular surround of the same mean luminance and chromaticity. Both were superimposed on an 18° diameter 565-nm background intended to suppress rods.

In the lower panels, the contrast sensitivities shown in the upper panels have been replotted as amplitude sensitivities (the flicker amplitude is simply the contrast multiplied by the mean level). Such plots make it easier to visualise conditions under which amplitude sensitivity is roughly independent of the mean light level, and, indeed, at higher frequencies the amplitude sensitivities appear to converge along a common high-frequency asymptote. Consequently, the speeding up of the visual response with intensity has been thought to keep *amplitude* sensitivity at higher flicker frequencies approximately independent of mean level (until, at the very highest light levels, photopigment bleaching becomes important and causes frequency-independent sensitivity losses). However, when the same data are plotted against linear frequency as in Figs 2–6, below, it is evident that the convergence of the high frequency sensitivity is at best approximate. This approximate “amplitude-invariance” has been inappropriately named “high-frequency linearity” [15]—inappropriate because changes in the time constant and gain with mean level make the underlying system inherently nonlinear. One reason that the notion of amplitude-invariance has persisted in spite of clear evidence to the contrary [13] is that when TCSFs are plotted on a logarithmic rather than linear frequency axis, the sensitivities become increasingly compressed at higher frequencies (compare Fig 1 with the same data shown in Figs 2 and 3), giving the impression of convergence.

Other characteristic properties of the TCSF that can be seen in both the upper and the lower panels of Fig 1 are that at low mean levels the TCSF shapes are typically “low-pass” in form; that is, sensitivity decreases monotonically as frequency increases. In contrast, at medium and high intensities the TCSF shapes are “band-pass” in form; that is, sensitivity is greatest at some intermediate frequency and decreases as frequency is increased or decreased from that of the peak sensitivity. Additionally, the frequency of peak sensitivity increases with increasing mean level. As we discuss below, the change from band-pass to low-pass as light level deceases may reflect rod involvement in some of the measurements.

In this paper, we propose a simple model that accounts for these and other features of light adaptation seen in TCSF data. To derive the model, we have used a set of classic and more recent TCSF data that are plotted as amplitude sensitivities in Figs 2–6. The more extensive data shown in Figures 2, 3, 4 and 5 are from De Lange [14], Kelly [15], Roufs, [17], and Stockman, Langendörfer, Smithson & Sharpe [13], respectively. The more restricted data shown in Fig 6 are from Rovamo, Raninen & Donner [18], Swanson, Ueno, Smith & Pokorny [19], and von Wiegand, Graham & Hood [20]. In Figs 2–6, the TCSFs are plotted against a linear rather than a logarithmic frequency axis to illustrate the exponential loss of sensitivity that occurs with increasing frequency in the high-frequency region. Each panel shows the results for a single observer viewing sinusoidal stimuli that flicker around a series of mean intensity levels. The significance of the solid red and black and dashed yellow lines will be explained below. We consider the relation between the model we have developed and related models in the Discussion.

## Methods

The data sets are historical, and the data plotted in Figs 1–6 have been extracted from the original publications of De Lange [14], Kelly [15], Roufs, [17], Raninen & Donner [18], Swanson et al. [19], and von Wiegand et al. [20]. The original data from Stockman et al. [13] were provided by one of the authors. The extracted and original data can be found in S1 Dataset. We have not been selective in our choice of data. The experimental conditions, which vary widely from laboratory to laboratory, are summarized in Table 1 and briefly described in the figure legends.

**Table 1:** Experimental conditions. Values in degrees are the diameters of the circular targets or backgrounds. Illuminance ranges are in units of log_10_ trolands.

Study	Target	Background/ Surround	Illuminance range	Notes
De Lange [14]	2° white.	60° matching surround.	-0.43–4.00	Poor luminance control at lower luminance.
Kelly [15]	50° white, vignetted from 50 to 68°.	None.	-1.22–3.97	
Roufs [17]	1° “practically” white.	None.	0.30–4.40	
Stockman *et al*. [13]	4° 610-nm.	9°, 540-nm background.	0.42–5.69	Protanopes. Radiances chosen so that the maximum M-cone contrast is 13%.
Rovamo *et al*. [18]	1.66°, white.	3.32°, matching white surround.	-0.70–3.40	
Swanson *et al*. [19]	2° red and green LEDs metameric with 600-nm light	None.	-0.05–2.95	
von Wiegand *et al*. [20]	1° falling to zero intensity at 2° with a cosine function, 625-nm.	Matching 18° annular surround, all on an 18° 565-nm background.	0.64–3.64	

Model fitting was carried out using nonlinear regression implemented in SigmaPlot (Systat Software, San Jose, CA) based on the Marquardt-Levenberg algorithm [21, 22] that minimizes the sum of the squared differences between predictions of the model and the data.

### Data analysis and modelling

#### Exponential high-frequency sensitivity losses

Figs 2–6 show the sets of TCSF data from twelve individual observers that we have used to develop our light adaptation model. Unusually, we have plotted the logarithmic sensitivities as a function of linear frequency to emphasise an important characteristic of TCSF data: in the high frequency region, temporal sensitivity falls *exponentially*. Consequently, high-frequency sensitivity in Figs 2–6, where log sensitivity is plotted against a linear frequency scale, follow the straight lines shown by the red lines fitted to the higher-frequency data from each TSCF. The fits were of the form:

$$\log_{10}(A(f))=\frac{f}{m}+c$$ (1)

where *A(f)* is the amplitude sensitivity, *f* is frequency, *1/m* is the slope of the line and *c* is its intercept. A crucial concern was to determine objectively the lower limit of the frequency range over which it is reasonable to fit a line given by Eq (1). For the TCSF data in Figs 2–4 and 6, the limits were determined by making repeated least-squares fits that extended in steps from high to low frequencies until the added data point lay clearly outside the 95% confidence bands of the fit. For the TCSF data in Fig 5, for which we have the standard errors of the original measurements, the fits were extended in steps from high to low frequencies until the potentially added point lay more than two standard errors away from the fitted line.

The results of the fits are tabulated in Table A in S1 Appendix, which gives values for the fitted parameter (*m*), the standard errors for each fit, and the *R*^*2*^ values. As can be seen in Figs 2–6, the straight-line fits for all twelve observers are generally excellent over substantial frequency ranges. *R*^*2*^ values varied between 0.917 and 0.999 with a median *R*^*2*^ of 0.987. The data strongly support a linear relation between log_10_ sensitivity and frequency and thus show that, in the high-frequency region, flicker sensitivity declines exponentially with increasing frequency.

**Fig 7:**
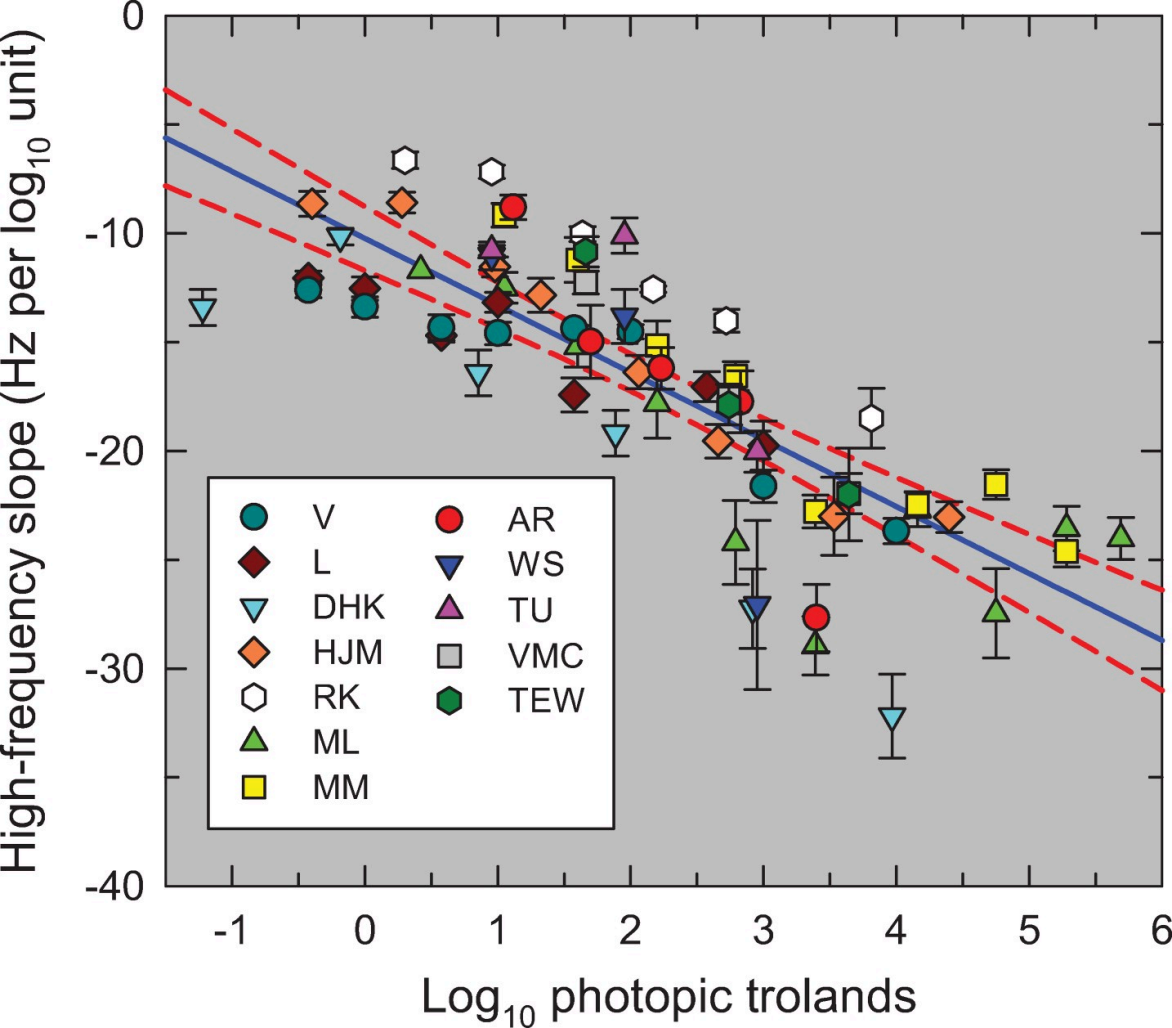
The reciprocal of the slopes of the red lines in Figs 2–6 (Hz per log_10_ unit sensitivity) plotted as a function of the mean intensity in log_10_ photopic trolands. The lines were fitted to the higher frequency amplitude-sensitivity data for each observer for each mean light level. The error bars are ±1 standard error of the fitted slope (see Table A). The solid blue line is the linear regression (Pearson correlation coefficient = 0.801, p<0.0001) and the dashed red lines are the 95% confidence intervals.

Fig 7 shows *m* (in Hz per log_10_ unit of sensitivity)—the reciprocal of the best-fitting high-frequency slopes—as a function of light level (log_10_ photopic trolands) for all the TCSFs. We plot the slopes unconventionally in Hz per log_10_ unit for two reasons: first, because there is an approximately linear relation between the slope in this form and log light level as shown by the solid blue line in Fig 7 (Pearson correlation coefficient = 0.801, p<0.0001); and second, because of the simple relation between slope in this form and corner frequency (a crucial parameter in the model that is related to the time constant). Fig 7 shows that the high-frequency slopes of the TCSFs decrease with increasing light level.

The observation that TCSFs follow an exponential function at middle to high frequencies has been noted before [23, 24], and has been evident when TCSFs are occasionally plotted as a function of linear frequency (e.g., Figures 10 and 11 in [13]). However, both Kulikowski [23] and Watson & Ahumada [24] assumed that the slope on these co-ordinates was independent of light level, which is clearly not the case. The decrease in the high-frequency slope with mean level seen in Fig 7 is also contrary to the idea that the high-frequency TCSF slopes reach a common asymptotic slope that is independent of light level—the customary assumption associated with “high-frequency linearity” or as we refer to it "amplitude-invariance", which is not supported by the data.

The exponential fall-off with a rate of loss that decreases with light level puts an important constraint on the form of any light adaptation model.

### Low-frequency sensitivity changes

In addition to accounting for the exponential high-frequency sensitivity losses, any light adaptation model must also account for the low-frequency sensitivity loss or attenuation that causes the TCSFs to be band-pass in shape, and it must also account for the changes in low-frequency sensitivity with adaptation, which are generally assumed to follow Weber's Law. According to Weber’s Law, the ratio of threshold intensity to the background intensity (*ΔI/I*) is constant. Thus, if Weber’s Law holds, the contrast sensitivity (*I/ΔI*) should be fixed, and the amplitude sensitivity (1/*ΔI*) should decrease as *I* increases. Thus, for TCSFs plotted as log_10_ amplitude sensitivities, if Weber’s Law holds changing the mean background from *I*_1_ to *I*_2_ should shift those sensitivities vertically by log_10_(*I*_2_/*I*_1_). Although de Lange’s observer V and Kelly’s observer DHK shown in Fig 1 obey Weber’s law at low frequencies over much of the intensity (see Fig 1), the data for other observers are less convincing. Examples of only approximate obedience to Weber’s Law include de Lange’s observer L (see Figure 6 in [14]), Stockman *et al*.’s observers MM and ML (see Figure 3 in reference [13]), von Wiegand *et al*.*’s* observers VMC and TEW (see Figure 2 in [20]), and Roufs’ observers HJM and RK.

### A simple model of light adaptation

In this section, we construct a model to account for the TCSFs at both low and high temporal frequencies. A classic approach to modelling human flicker sensitivity is to envisage the visual pathway as a filter made up of a cascade of simpler constituent filters, with or without feedback or feedforward inhibition, and to assume that the output of the cascade reflects sensitivity (e.g., [18, 25, 26–28]). The simple filters that make up the cascade are typically assumed to be leaky integrators with outputs that step up and decay exponentially in response to a brief pulse at their input. Leaky integrators, which are often associated with neural processes in the visual system [29], are characterized by their time-constants. The time-constant (usually denoted by τ) is simply the time taken for the output to fall to 1/*e* (36.79%) of its peak value following a brief pulse at its input. Leaky integrators, which are also known as “RC filters” in electronics, behave as low-pass filters with a monotonically decreasing response to sinusoids of increasing frequency. Such filters can also be characterized by their cut-off or corner frequency, *f*_*c*_—the frequency at which the response amplitude has fallen by a factor of √2 (or 0.15 log_10_ units) below its maximum. The corner frequency and time-constant are inversely related, $\tau =1/\left(2\pi f_{c}\right)$. We prefer to use corner frequency because it is simpler for characterizing data, like those in Figs 1–6, that are presented in the frequency domain.

Leaky integrators are also equivalent to first-order chemical reactions (*e*.*g*., a substance A decomposing to substance B with a time-constant τ). Networks of leaky integrators therefore provide plausible models of the cascades of biochemical and/or neural processes in photoreceptors and neural pathways. To avoid repetition, we will simply call each leaky integrator element of the cascade a "low-pass filter stage" or "LP-stage" for short. Light adaptation is primarily achieved by increasing the corner frequency (or equivalently shortening the time-constant) of some or all of the LP-stages in the cascade that mimics the visual response. As we develop below, the low-frequency attenuation or high-pass filtering seen in the data can be implemented biochemically or physiologically by feedback or by parallel feedforward pathways.

**Fig 8:**
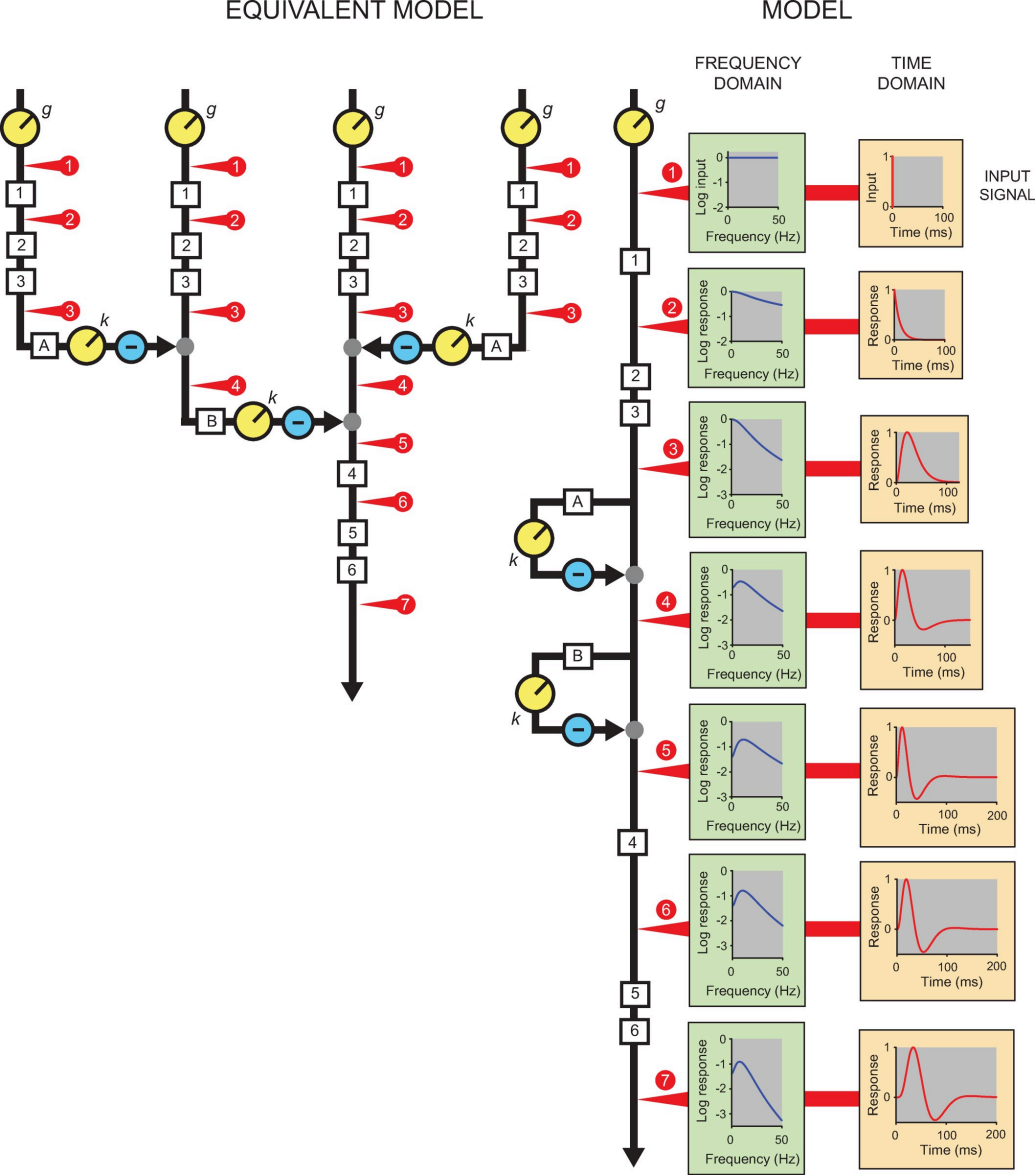
The direct cascade used to model flicker sensitivity is shown along the vertical black line labelled MODEL and has six low-pass stages (1–6) and two inhibitory stages (A and B). The six variable speed LP-filters (1–4, and A, B) have the same corner frequency of 15 Hz in this example and the two fixed-speed LP-filters (5, 6) have a corner frequency of 30 Hz (see text). The effects on an impulsive input signal (top orange panel) at different stages of the model (denoted by the numbered red arrows) are shown as a function of time in the orange panels of right-hand column and, in the corresponding green panels, as the logarithm of amplitude of the response as a function of frequency. The amplitude spectrum of the input is flat with equal amplitude at all frequencies (top green panel). An early gain adjustment, *g*, controls the overall gain of the system. The second orange and green panels show the effects of the first LP-stage and the third orange and green panels, the effects of a cascade of three stages. Next, the fourth and fifth pair of panels show the additional effects of one and two feedforward stages, respectively. The two feedforward stages include a common scaler, *k*, after which the feedforward signal is subtracted from its input. They produce a triphasic temporal response (orange panels) and a bandpass frequency response (green panels). The sixth pair of panels show the effects of another LP-stage with a corner frequency of 15 Hz and the final pair the effects of two final stages with corner frequencies of 30 Hz. The Equivalent Model on the left is mathematically equivalent to the cascade but has lateral connections that are more consistent with other psychophysical and physiological data.

Fig 8 illustrates the model and how a brief pulse is transformed as it passes through it. The cascade is indicated by the black line labelled MODEL and is a cascade of six LP-stages (1–6) and includes two feedforward stages (A and B) that each incorporate a further LP-stage. The order of the components shown here is essentially arbitrary because the order of linear elements in a cascade has no effect on the final output (i.e., on the TCSFs), but we justify this configuration below based on a review of physiological studies. In this illustration, the corner frequencies of the six variable speed LP-filters (1–4, and A, B) are the same (*f*_*c*_ = 15 Hz); the two fixed-speed LP-filters share a different corner frequency (*f*_*cL*_ = 30 Hz). (These values are consistent with the model fits explained later.) The Equivalent Model on the left is more plausible physiologically but is mathematically identical if the inputs to each of the four streams are the same (see Discussion).

The right-hand column of orange panels illustrates the temporal response at various points in the processing stream to the brief input pulse that is shown in the top orange panel. The temporal responses (each normalised to unity), are plotted as a function of time—that is, they are displayed in the “time-domain”. The column of green panels illustrates the corresponding amplitude responses at the corresponding points in the stream to the same brief input pulse. The logarithm of the amplitude responses, plotted as a function of frequency, are in the “frequency-domain”, as are the flicker-sensitivity measurements of Figs 2–6. As illustrated in the top green panel, the amplitude spectrum of the input pulse is flat with the same amplitude at all frequencies. The numbered red arrows indicate the locations of the responses shown in the corresponding green and orange panels.

The second row of panels show the response of a single LP-stage—an exponentially-decaying response in the time-domain and a low-pass frequency response in the frequency-domain. The third panels show the response after processing in three LP-stages. The response is delayed and smeared out over time in the time-domain but still has a low-pass frequency response albeit with a more steeply falling high-frequency slope.

Although cascades of LP-stages can capture the high-frequency characteristics of TCSFs, they cannot give rise to the loss in sensitivity at low-frequencies that causes the TCSFs at middle- and high-light levels to be bandpass. To produce low-frequency attenuation, we have added two inhibitory feed-forward stages between the third and fourth LP-stages. The inhibitory stages act by passing their visually-derived input signal through separate LP-stages (A and B) before scaling the resulting signal (by *k*) and subtracting the result from the original signal. For *k* = 1 these are standard high-pass filters, while for 0<*k*<1 these feedforward stages are commonly known as “lead-compensator” filters in engineering because they introduce a phase advance (or lead) that can improve the stability of the system. As shown in the corresponding orange panels, the inhibitory stages advance the peak, sharpen the temporal response and, at some point, bring the temporal response below zero (a “biphasic” response). They also cause a later, small positive response, so that the temporal response is actually “triphasic” [30, 31]. We consider the origin of the biphasic response in the Discussion. The corresponding green boxes show that the frequency-domain effect of the inhibitory stages is to produce low-frequency attenuation. The fifth orange and green panels show the effects of adding another variable-speed LP-stage and the sixth pair of panels the effects of adding two fixed-speed LP-stages.

We have chosen the sequence shown in Fig 8 based in part on other work in which we dissected the visual pathway into an early bandpass filter with variable corner frequencies and a later lowpass filter with two LP-stages with fixed corner frequencies [32–34].

The Equivalent Model shown on the left is identical to the standard Model when its four inputs are the same as the single input in the standard model. The numbered red circles and arrows show corresponding points in the two versions. The Equivalent Model can be thought of as showing the four possible “routes” through the original Model (including routes through either or both feedforward stages). Note that the Equivalent Model can be modified to allow differences between the four inputs, thus extending the model into the spatial domain. The Equivalent Model with parallel pathways is more consistent with known retinal physiology and with lateral interactions mediated, for example, by horizontal cells between nearby cones [35]. Because inhibition is derived from lateral connections in the Equivalent Model, variations in *k* will produce spatial effects on the temporal response to flicker and brief flashes. However, the subtractive inhibition need not necessarily involve lateral interactions, and may instead involve self-cancellation in longitudinal inhibition of the direct pathway.

The final form of the model was chosen partly on the basis of a series of preliminary fits. These showed that across all datasets at least six LP-stages are required in the direct cascade to account for the high-frequency slopes of the TCSFs (although a greater number of LP-stages each with higher corner frequencies would also give good fits). Similarly, a minimum of two inhibitory stages are required to account for low-frequency attenuation (although a greater number of inhibitory stages with lower values of *k* would also give good fits). An important simplifying assumption was that the variable corner frequencies of the first four LP-stages in the cascade and the two LP-stages embedded in the inhibitory stages are the same and that they all varied together with mean light level. This seems an unlikely assumption but allowing the corner frequencies to vary independently added many more model parameters without significantly improving the fits.

A second simplifying assumption was that the best-fitting corner frequencies of the two fixed-speed stages, *f*_*cL*_, are the same, and that they do not vary across observers. When *f*_*cL*_ was allowed to vary across observers in preliminary fits, the best-fitting values were generally similar and had large standard errors, so that fixing *f*_*cL*_ across observers resulted in only a minimal reduction in the quality of the fits.

The third simplifying assumption was that for each observer the gains of the two feed-forward stages (*k*) are the same and are independent of light level. This assumption was also based on preliminary fits, which showed that *k* did not vary significantly across medium and high light levels. The reduction in *k* that is found at low light levels may be due to light-dependent changes in *k* within a single mechanism, but it might also be due to the contribution of rods at mesopic levels. Because rods are more sluggish than cones (e.g., [36]), their contribution is likely to increase sensitivity at low frequencies and thus will have the effect of reducing *k* in the fits. To avoid the potentially confounding effects of rods, we have excluded TCSFs from the analysis and modelling that are likely to depend on detection by both rods and cones. The levels assumed to be cone-mediated are highlighted in yellow in the keys of Figs 2–6; the rationale behind those choices is described in more detail in S1 Appendix. In general, mean levels below about 1 log_10_ photopic trolands were excluded from the main analysis unless the experimental conditions excluded rod detection.

These assumptions led to a simple model with just four parameters of which two, *f*_*c*_ and *g*, vary with light level, and two others, *k* and *f*_*cL*_, do not. The model is defined by Eq (2):

$$A\left(f\right)=g\times {\left(\frac{1}{\sqrt{f^{2}+f_{c}{}^{2}}}\right)}^{4}\times {\left(\frac{\sqrt{f^{2}+{\left(\left(1-k\right)f_{c}\right)}^{2}}}{\sqrt{f^{2}+f_{c}{}^{2}}}\right)}^{2}\times {\left(\frac{1}{\sqrt{f^{2}+f_{cL}{}^{2}}}\right)}^{2}$$ (2)

where *A(f)* is the amplitude sensitivity, *f* is frequency (Hz), *g* is the overall gain, *f*_*c*_ is the corner frequency of the six variable-speed LP-stages (Hz), *k* is the common gain of the two inhibitory stages, and *f*_*cL*_ is the corner frequency of the two fixed-speed LP-stages (Hz). [The corner frequency equals 1/(2πτ), where τ is the time constant in seconds.] The terms of Eq (2) have been grouped so that the first term is the frequency-independent gain, the second term corresponds to the four variable LP-stages, the third term corresponds to the two lead-compensators, and the final term corresponds to the two fixed LP-stages. We discuss the critical aspects of the model assumptions more fully in the Discussion. Other equations characterizing the model are provided in S1 Appendix.

The results of the fits are tabulated in Table B in S1 Appendix and the fits are shown by the solid black and dashed yellow lines in Figs 2–6. The black lines show the fits at levels assumed to be photopic (cone-mediated). For these fits, as just noted, *k* was fixed for each observer and did not vary with light level. The dashed yellow lines show the fits at levels assumed to be mesopic (rod-and-cone-mediated). For these fits, *k* was allowed to vary with light level for each observer. As can be seen, the model does a remarkably good job of accounting for the data at all levels—both mesopic and photopic. In the remainder of the paper, however, we consider only the fits at photopic levels. The adjusted *R*^*2*^ value for our model for cone-mediated (photopic) levels is 0.996.

**Fig 9:**
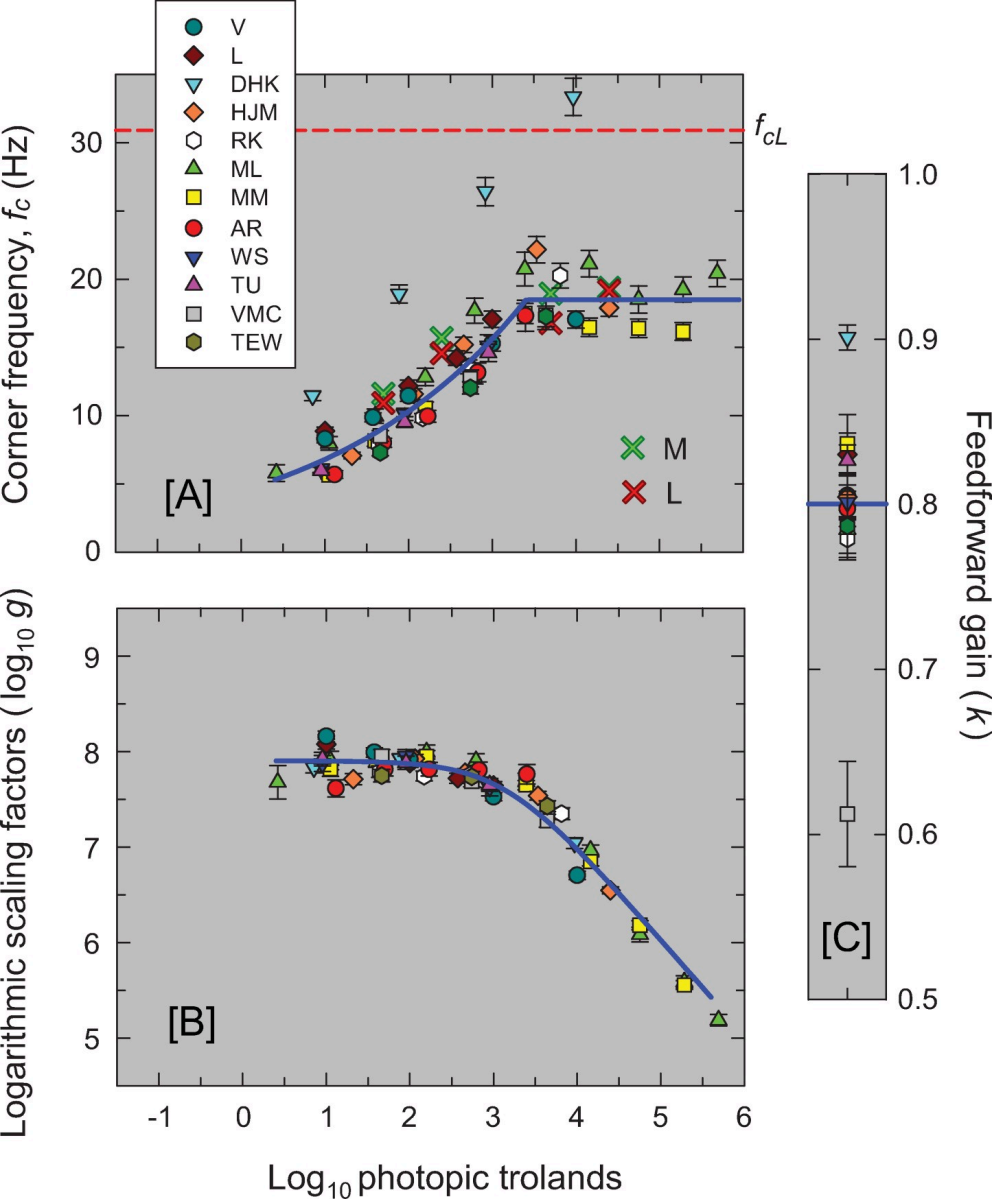
The three panels show the model’s best fitting parameter values for each of the twelve observers. The error bars are ±1 standard error of the fitted parameter. Panel [A] shows the best-fitting corner frequencies (*f*_*c*_) in Hz common to the six variable-speed LP-filters as a function of mean level (log_10_ photopic trolands). [Corner frequency (Hz) is inversely proportional to the time constant τ: *f*_*c*_ is equal to 1/(2πτ) where τ is in seconds.] The solid blue line through the data is the descriptive standard or mean function defined by Eq (3). The dashed horizontal red line marks the constant corner frequency of the final two fixed-speed stages (30.9 Hz). The red and green crosses are the corner frequencies of a 3-stage LP filter fitted to primate L- and M-cone responses, respectively, measured by Baudin *et al*. [37] and shown in Fig 10. Panel [B] shows the logarithmic of the best-fitting overall gain, *log*_*10*_*g*, but with the data for each observer vertically shifted to align with the descriptive standard function shown by the solid blue line and defined by Eq (4). Panel [C] shows the best-fitting scaling factor, *k*, common to the two inhibitory feedforward stages and fixed across mean luminance levels. The horizontal solid blue line shows the mean value of *k* (0.80). Only the parameters for levels thought to be cone-mediated are shown.

The best-fitting parameters for cone-mediated vision are plotted for all the observers in Fig 9. Panel [A] shows the best-fitting variable-speed corner frequencies (in Hz) as a function of mean level for each observer as noted in the figure key. The best-fitting corner frequency (30.9 Hz) for the two fixed-speed filters common to all observers is shown by the dashed red line. Panel [B] shows the best-fitting overall gains, *log*_*10*_ *g*, vertically aligned with the average gain captured by the blue line about which the values for each observer cluster closely. (The unshifted values of *log*_*10*_*g* are given in Table B in S1 Appendix.) Panel [C] shows *k*, the best-fit for the feedforward inhibition. The value of *k* is fixed across levels for each observer.

The corner frequency and gain parameters agree well across observers with one easily explicable exception. The unusually high parameter values of corner frequency and *k* for DHK (light blue inverted triangles) from Kelly [15] probably reflect the use of a very large flickering target (50°in diameter vignetted to zero intensity by 68°); the other studies used smaller targets of between 1 and 4° in diameter. With hindsight, the large target size was probably unfortunate because temporal sensitivity is highly inhomogeneous across the retina (e.g., [38]). Although the potential effects of retinal inhomogeneity are not obvious in that classic data for DHK shown Figs 1 and 3, the TCSFs for other observers from the same study are much more irregular [39].

At low to moderate intensity levels, changes in low-frequency sensitivity in our model are accounted for by changing the corner-frequencies of the LP-filters in our model with little or no change in gain. Unlike other models (e.g., [17, 27, 28]), a change in the strength of the feedforward or feedback signal is not required.

### Standard model parameters

To standardise the model parameters for a typical observer, we next derived descriptive functions to quantify the dependence of corner frequency (*f*_*c*_) and overall gain (*g*) on mean light level (in log_10_ photopic trolands). Values from the standard formulae are shown by the blue lines in Panels [A] and [B] of Fig 9. For *f*_*c*_, we, adopt a simple function that grows as a power law until it asymptotes at an upper limit for *f*_*c*_. The form of this function is:

$$f_{c}=\min(aI^{b},\;d)$$ (3)

where *f*_*c*_ is the corner frequency in Hz, *I* is the mean background level (retinal illuminance in photopic trolands), *a* is a constant, *b* an exponent, *d* is the upper limit for *f*_*c*_, and the operator min() means "the lesser of" its arguments (*aI*^b^ or *d*). The best-fitting values for the function fitted to the *f*_*c*_ parameters for all observers (except for DHK—for the reasons discussed above) are *a* = 4.48±0.35, *b* = 0.181±0.01 and *d* = 18.49±0.42. The fit, shown by the solid blue line in Panel [A] of Fig 9, has an adjusted *R*^*2*^ of 0.89. A power function, but without an upper limit, was also adopted by Rovamo *et al*. [18] based largely on a review of photoreceptor and other measurements by Donner *et al*. [40].

We adopted the following equation to describe the dependence of the logarithm of the scaling factor, log_10_ *g*, on the mean background retinal illuminance, *I* (Panel [B]):

$$\log_{10}g=-\;\log_{10}(I+I_{0})+s$$ (4)

where *I*_*0*_ is constant across all observers and *s* takes on a different but constant value for each observer. *I*_*0*_ indicates the mean luminance below which log_10_ *g* is approximately constant and above which a plot of log_10_ *g* vs log_10_ *I* eventually approaches an asymptotic slope of -1 (as in Fig 9B); *s* just shifts the function for each observer vertically without changing its shape. The best fitting value of *I*_*0*_ is 10^3.13±0.06^ and the *R*^*2*^ value for the fit is 0.973. The mean value of *s* across all 12 observers is 11.03. Panel [B] of Fig 9 shows the individual values of log_10_*g* vertically aligned with the function given by Eq (4) with *s* set to the mean value of 11.03.

The mean value for the feedforward gain, *k*, is 0.80—as indicated by the horizontal blue line in Panel [C] of Fig 9. Eqs (3) and (4), with *f*_cL_ = 30.9 and *k* = 0.80, can be used with Eq (2) to calculate the expected amplitude sensitivity as a function of frequency for any mean retinal illuminance between 0.4 and 5.7 log_10_. They can also be used with Equation (B) in S1 Appendix to calculate the triphasic temporal response of the system to an impulse; i.e., with a positive lobe, a negative lobe, and a second much smaller positive lobe (see lowest orange panel of Fig 8).

The parameters plotted in Fig 9 suggest that shortening the integration time is important for sensitivity regulation up to about 3.1 log_10_ trolands after which sensitivity regulation depends primarily on frequency-independent decreases in the overall gain. Decreases in gain above about 4.3 log_10_ trolands are likely due to photopigment bleaching (e.g., [41]), which, by reducing photon capture at the input, effectively reduces the gain of the system and so helps prevent the cone-mediated system from saturating (see Figure 9 in reference [42]). However, the decrease in gain between 3.1 and 4.3 log_10_ trolands must be due to other sensitivity-regulating mechanisms that attenuate the visual response in an approximately frequency-independent manner. These mechanisms might also include shortening the integration times of additional LP-stages with corner frequencies near or above the temporal acuity limit (*i*.*e*., with *f*_*c*_ > 80 Hz), so that at lower visibly flickering frequencies, their effect on the TCSF is much like a change in overall gain.

## Discussion

We have proposed a simple model of human light adaptation made up of a cascade of low-pass filters and two stages of subtractive inhibition. Sensitivity regulation depends on two intensity-dependent parameters: one that controls the speed of the response through a single corner frequency, *f*_*c*_ (or time constant, τ) and another that, over a limited upper intensity range, controls the size of the response through the gain parameter, *g*. The model provides excellent predictions for 65 years of existing TCSF data from De Lange [14], Kelly [15], Roufs [17], Stockman *et al*. [13], Rovamo *et al*. [18], Swanson *et al*. [19] and von Wiegand *et al*. [20] as shown in Figs 2–6. We consider the crucial Physiological basis of the model below.

### Essential and non-essential features of the model

We readily acknowledge our debt to previous work on light adaptation and to previous models of light adaptation, many of which incorporate, in different configurations, the model elements used here (e.g., [14, 15, 18, 26–28, 43–49]). Helpful, insightful reviews of light adaptation include those by MacLeod [50], Geisler [51], Hood & Finkelstein [7], Laughlin [52], Graham & Hood [53], Hood [54], van Hateren [55] and van Hateren & Snippe [56].

Our simplified model is made up of a direct cascade of six LP-stages and two inhibitory stages, each of the latter having a gain control, *k*, and an embedded LP-stage. As we have shown, we can provide an excellent description of existing TCSF data by adjusting the time constants of the four earliest LP-stages in the direct path and the two in the inhibitory stages with light level and, at higher levels, the overall gain, *g*, but fixing the strength of inhibition, *k*, and the time constants of the final two LP-stages. An important question, then, is which features of the model are essential to its success and which can be changed without upsetting the model's predictions? Answering this question will help place the model within a wider physiological context and help us to relate it to previous models.

First, the numbers of LP-stages and inhibitory stages are poorly constrained by the data. This uncertainty is evident in the variability in the number of LP-stages used in earlier models(e.g., [14, 18, 27, 28, 45–49]). In general, we found that reducing the total number of variable- and fixed-speed LP-stages below six, or the number of inhibitory stages below two, started to lower the goodness-of-fit, but increasing them, with compensatory increases in the corner frequency or reductions in *k*, had relatively little effect. We therefore regard six and two as lower bounds on the number of direct LP-stages and inhibitory stages, respectively, but accept that there could be more.

Second, the way in which the corner frequencies of the LP-stages change with light level, and how many of them change are also both poorly constrained. Based on other evidence from our laboratory, which suggests that one or two “central” LP-stages have fixed corner frequencies that do not change with light level [34, 57], we have chosen to fix the corner frequencies of the final two of the six LP-stages in the direct cascade. However, we found that the model predictions are only slightly worse if we fixed the corner frequencies of between zero and four LP-stages in the direct cascade. A relevant consideration is that the number of fixed stages affects the dependence of log_10_*g* on luminance (see Fig 9B). If the corner frequencies of zero or one LP-stages are fixed, log_10_*g increases* with luminance at the lower background levels (i.e., the gain is reduced at low luminances). By contrast, if between 2 and 4 LP-stages are fixed, log_10_*g* remains roughly constant at low levels (as in Fig 9B). Although comparable decreases in gain have been suggested before [13, 18], an increase in log_10_*g* is counter to the prevailing view of light adaptation as a way of reducing gain as light level increases (the so-called dark-glasses model, see [50]). We therefore chose to have two fixed stages, which obviates the need to account for counterintuitive decreases in gain at low light levels.

Third, the assumption that the variable corner frequencies all vary together is almost certainly a simplification. While there are some advantages in having corner frequencies that change together (*e*.*g*., it produces maximum sensitivity at all frequencies, see S1 Appendix), it seems unlikely that they will all change together and in the same manner. The assumption that the LP-filters in the inhibitory circuits are the same as those in the direct cascade also seems unlikely. However, to be consistent with the low-frequency attenuation and the peaks found in the bandpass TCSF data, the corner frequencies in the inhibitory circuits must be above about 8 Hz, and thus are comparable to the corner frequencies of the LP-filters in the direct cascade.

Last, we assumed that the strength of subtractive inhibition does not change with light level; *i*.*e*., that *k* is constant in our model. Note that, as we discuss further in the next section where we consider the physiological basis of the model, this subtractive inhibition is most likely to be mainly mediated by lateral connections, rather than being inside the photoreceptor. The fixed value of *k* is seemingly at odds with the general view that TCSFs are low-pass in the dark and band-pass at higher light levels, but the conditions under which the low-pass TCSFs were measured were mesopic, so that those TCSFS are likely to be influenced by rod intrusion. Thus, most low-level TCSFs—with some exceptions—cannot be considered as representative of the same system that governs high light-level TCSFs. A similar conclusion was reached by Rovamo *et al*., who incorporated a high-pass filter in their psychophysical model. Their high-pass filter was also fixed with light level, a decision they supported by appealing to cat ganglion cell data that showed little change in the surround strength with light level [58, 59]. There is also good psychophysical evidence that photopic spatial integration changes little with light adaptation [60]. The crucial difference between our model and that of Rovamo *et al*. is the form of the high-pass filter, which in their case was a differentiator with a 1/ *f* attenuation characteristic. This predicts that, on a double-logarithmic plot, as in, for example, Fig 1, log sensitivity should increase in direct proportion to log frequency (i.e., it should be a straight line with unity slope), whereas the data (other than for mesopic levels) consistently show an accelerating increase in sensitivity up to about 5 Hz.

In summary, the essential features of the model are a direct cascade of at least 6 LP-filters, the corner frequencies of some or all of which change with light level; and at least two stages of subtractive inhibition the gains of which do not change with light level. In addition, an overall light-dependent change in gain is required at higher light levels.

### Physiological basis of model

Our model is essentially agnostic as to the locations and order of the model elements within the visual pathway, which as we noted above could be in any order. Psychophysical measurements generally do not allow us to locate the model elements with certainty, with some exceptions. For example, the appearance of very-high spatial frequency gratings produced by laser interferometry suggests that adaptation occurs within single cones [61]. We have nevertheless placed the elements in Fig 8 in a particular order, based in part on physiological measurements that we consider next. Links between physiology and psychophysics, however, are inevitably tentative, and, as we discuss next, the nature of some of the physiological measurements makes them unlikely to be representative of normal visual function. We start with measurements of photoreceptor adaptation.

#### Photoreceptors

As we argued in the Introduction, for light adaptation to protect the neural pathways from saturation at moderate to high light intensities much of the sensitivity regulation should occur within the photoreceptor at its outer segment, inner segment and/or pedicle. Thus, we should expect clear evidence for at least *some* of the variable-speed LP-stages from our model in primate photoreceptor measurements. Surprisingly, however, the available data are highly contradictory. The very influential primate suction-electrode data (in which cone outer segments are drawn inside suction electrodes and their current responses to light recorded) suggest that cone adaptation does not reduce sensitivity by 50% until the background intensity reached as high as 3.3 log_10_ td (see Figure 8 in [62]). [We refer to background that reduces sensitivity by 50% as *I*_*0*_—as in Eq (4)]. Moreover, the photocurrent responses show minimal changes in speed over the entire background-intensity range, which suggests that there are *no* variable LP-stages in the cone outer segment. These results seem implausible and suggest, as others have pointed out (e.g., [63]), that the separation of the outer segment from the retinal pigment epithelium (RPE) and from its normal extracellular environment, results in unnatural flash responses. This argument is bolstered by the fact that the photocurrent responses are far too sluggish to support the cone frequency responses estimated from psychophysics (see, for example, Figure 12 in [64])—in contrast to the faster photovoltage measurements of cone inner segments made by the same group [65]. Yet, although those photovoltage measurements are faster, the average *I*_*0*_ was still as high as 2.8 log_10_ td, and the backgrounds still did not substantially change the kinetics of the cone response (see Figure 12A in [65]). Together, these results implausibly suggest that there are no kinetic changes in the cones and that little adaptation of any type occurs below 2.8 log_10_ td.

At the time, the lack of cone adaptation seemed consistent with extracellular mass recordings of cone responses to 150-ms flashes made by Boynton & Whitten [66], who explained their data solely in terms of response compression and photopigment bleaching without active adaptation. In contrast, similar measurements, but using both incremental and decremental flashes, by Valeton & van Norren showed evidence for adaptation with an *I*_*0*_ of approximately 2 log_10_ td (see Figure 7 in [67]). Seipel, Holopigian, Greenstein & Hood [63] measuring focal electroretinograms (FERGs), which they argued reflect photoreceptor responses at frequencies above 20 Hz, showed clear temporal frequency-dependent sensitivity changes above 1.5 log_10_ td that were consistent with their human psychophysical measurements. These results are consistent with a speeding up of the visual response (see Figure 1 in [63]).

Dunn, Lankheet & Rieke [68] subsequently made current recordings of primate cones using perforated or whole-cell voltage clamps and showed that adaption begins to reduce cone sensitivity above about 1000 P/s (photoisomerization per second) with sensitivity falling to half of its dark value by 5500 P/s. These values are approximately 1.7 and 2.5 log_10_ trolands, respectively, so that the *I*_*0*_ value is only 0.3 log_10_ unit lower than the value estimated by Schneeweis & Schnapf [65]. Crucially, Dunn *et al*. [68], in contrast to the earlier work, reported that their cone responses clearly sped up with adaptation, thus providing the first direct evidence of such effects in primates, even given the provisos that the cones were separated from the RPE and that voltage-clamping obviates the effects of voltage-gated ion channels on the photoreceptor response [69].

**Fig 10:**
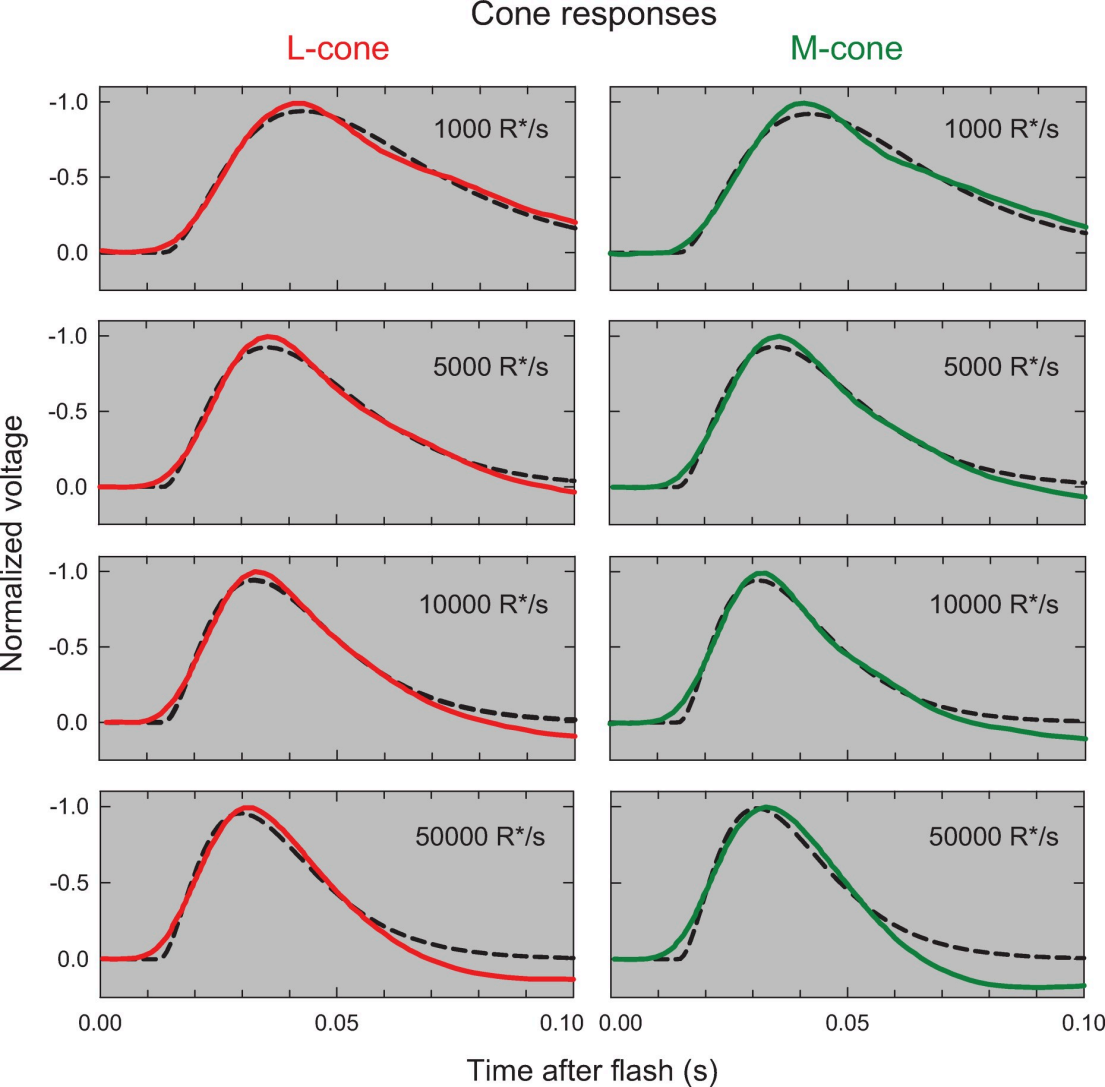
The panels in the left and right columns column show, respectively, the mean primate L-cone (red continuous lines) and M-cone (green continuous lines) responses measured by Baudin *et al*. [37] at mean levels of 1000, 5000, 10000 and 50000 photons absorbed per second (R*/s). The dashed black lines show the best-fitting 3-stage LP filter responses with common corner frequencies, which vary with light level. For further details see text.

Such inconsistent physiological results with *I*_*0*_ values that range from 1.5 to 3.3 log_10_ td and with evidence for and against cone responses speeding up with adaptation make it difficult to locate the elements of the psychophysical model inside or outside the photoreceptors. Fortunately, very recent photovoltage measurements of primate M- and L-cones obtained at four adaptation levels [37] can help to resolve these discrepancies. Fig 10 shows the mean L-cone (red continuous lines, left column) and M-cone (green continuous lines, right column) responses measured at mean light levels of 1000, 5000, 10000 and 50000 R*/s (where R*/s is the estimated rate of photon absorption per second). These levels correspond to 1.7, 2.4, 3.7 and 4.4 log_10_ td, respectively (assuming 1 td = 20 R*/s, see p. 17 of their paper). We have separately fitted the L- and M-cone photoreceptor responses with the impulse responses of a 3-stage LP-filter allowing the best-fitting corner frequencies to vary with light level with a common best-fitting initial response delay. The fits are shown by the black dashed lines in Fig 10. The best-fitting L-cone corner frequencies are 10.92±0.12, 14.58±0.16, 16.78±0.21 and 19.29±0.24 Hz for levels 1000, 5000, 10000 and 50000 R*/s, respectively, with an initial delay of 13.50±0.13 ms and an overall *R*^*2*^ of 0.974. The best-fitting M-cone corner frequencies are 11.60±0.16, 15.69±0.23, 18.95±0.30 and 19.26±0.28 Hz, respectively, with an initial delay of 14.36±0.16 ms and an *R*^*2*^ of 0.960. As can be see, the fits shown by the dashed lines very plausibly account for the rising and falling phases of the photoreceptor responses.

The best-fitting corner frequencies have been plotted as red and green diagonal crosses in Fig 9A, where the values agree well with our model predictions. Our main purpose in showing these fits is *qualitative*. The cone responses show that adaptation begins in the cone at 1.70 log_10_ troland (and perhaps lower had lower levels been measured), and then is controlled by a change in the corner frequencies of a cascade of LP-filters. The increase in speed is consistent with results from other species (e.g., [70, 71]). There are important caveats. The L- and M-cone responses measured by Baudin *et al*. are of peripheral cones rather than of foveal cones so that the LP-stages are likely to be faster than the stages inferred from foveal flicker measurements (e.g., [72]). Moreover, plausible fits can be obtained with more than 3 LP-filters with concomitant reductions in corner frequency, so that the recordings do not constrain the number of LP-filters. And, the cones in these measurements have been separated from the RPE, so they may be somewhat atypical.

Note that the measurements shown in Fig 10 and other recent work show that cone responses are monophasic or weakly biphasic [37, 73] rather than strongly biphasic as was found in the early suction electrode recordings [62]. If we accept the more recent measurements, then the subtractive feedforward in our model must occur after the inner segments but may occur at the cone pedicles. Indeed, the recent work of Kamar, Howlett & Kamermans [74] shows clearly that the cone response is modulated by signals from surrounding cones fed back through horizontal cells.

Taken overall, we can reasonably conclude from the physiological data that cone adaptation begins as low as 1.70 log_10_ and takes the form of a speeding up of the cone response. Below that level, the evidence for cone adaptation is weak.

#### Frequency-dependent and frequency-independent adaptation

Whereas changing the corner frequencies of LP-filters generally causes frequency-*dependent* changes in the cone response, other adaptation mechanisms cause frequency-*independent* changes in sensitivity. One such mechanism, which protects the cone from saturation at very high light levels, is photopigment bleaching. By reducing photon capture at the visual input, bleaching effectively turns down the gain of the system and so helps prevent the cone-mediated system from saturating. The usual formula for *p*, the fraction of unbleached pigment remaining on a steady background of *I* photopic trolands, is *p* = *I*_*0*_/(*I*+*I*_0_), where *I*_0_ is the background intensity that bleaches 50% of the photopigment, a value typically assumed to be 10^4.3^ trolands or 4.3 log_10_ trolands [41]. The effect of bleaching is incorporated in our model as part of the gain control, *g*, which precedes the LP-filters and dominates adaptation above about 4.3 log_10_ td (see Figure 9 in [42]).

Our modelling of the TCSF data suggests that there is an *additional* frequency-independent gain control at lower light levels, which is unlikely to be due to bleaching. This additional gain and bleaching combine to produce an effective *I*_*0*_ of 3.13. Notably, this frequency-independent gain control has essentially the same properties as the gain control identified in cone outer segments by Schnapf *et al*. [62], which had an *I*_0_ of 3.3 log_10_ td and the similar gain control identified in photovoltage recordings by Schneeweis & Schnapf [65], which had an *I*_0_ of 2.8 log_10_. The gain control associated with bleaching, which necessarily precedes other adapting stages, and the additional gain control are both incorporated in the early gain control, *g*, in the model of Fig 8, and jointly described by Eq (4).

Two further points are worth noting. First, mechanisms that seem to be frequency-independent across the range of visible frequencies could include the frequency-*dependent* speeding up of LP-stages but with corner frequencies well above the temporal acuity limit (*i*.*e*., with *f*_*c*_ > 80 Hz). Such a speeding up may underlie the mechanism in the outer segment with an *I*_0_ of 3.13 log_10_ td. Second, any frequency-independent mechanisms after the outer segment will contribute to Eq (4), but for simplicity are included in the model as a single gain control, *g*.

The importance of frequency-independent gain changes in light adaptation has been a subject of much debate. Early studies used flashed stimuli, measuring either detection thresholds in psychophysics or the peak of the impulse response in electrophysiology (e.g.,[66, 67]). We note that these methods can confuse gain changes with changes in integration time. For example, consider the impulse response, *y*, of 3 identical, cascaded LP-stages, each with corner frequency, *f*_c_, and with an overall gain of *g*,

$$y\left(t\right)=g\frac{t^{2}}{2}e^{-2\pi f_{c}t}$$ (5)

The peak of this monophasic response occurs when its derivative is 0 for some time *t*>0,

$$\begin{array}{l} \frac{dy}{dt}=\left(gt-\pi f_{c}gt^{2}\right)e^{-2\pi f_{c}t}=0\\ \Rightarrow t=\frac{1}{\pi f_{c}} \end{array}$$ (6)

As *f*_c_ increases the peak occurs at earlier times, and the peak response is:

$$y\left(\frac{1}{\pi f_{c}}\right)=\frac{g}{2\pi^{2}f_{c}{}^{2}}e\left(-2\right)$$ (7)

The peak response increases in proportion to *g* and decreases in proportion to *f*_c_^2^. Differentiating between changes in speed or gain is not possible with flash responses without additional information; e.g., the time to the response peak.

#### Horizontal cells

Other indirect evidence of cone adaptation comes from Smith, Pokorny, Lee & Dacey [75], who measured the flicker and flash responses of primate H1 horizontal cells as a function of the mean background intensity and used the data to model cone adaptation. They found clear evidence that sensitivity regulation in H1 recordings begins at levels as low as 1 log_10_ trolands and that it is achieved by a speeding up of the visual response, which they attributed to a speeding up of the cone response. Both these properties are consistent with our model and point to adaptation occurring in the cone photoreceptor. The primary change in speed in their adaptation model is achieved by increasing the corner frequency of a single LP-stage from on average 4.2 Hz at 1 log_10_ troland to 53.1 Hz at 3 log_10_ trolands. Several other details of their model, such as the second-order filter required to account for resonance in their H1 data, are more likely to reflect the H1 network responses (e.g., [76]) than cones.

#### Bipolar cells

An important piece of evidence about the location of the adaptation sites comes from Dunn, Lankheet & Rieke [68], who compared cone and bipolar cell responses in the same preparation. They found that the bipolar cells did not contribute significantly to adaptation and essentially followed the cone responses without additional adaptation.

#### Ganglion cells

Ganglion cells provide evidence about the adaptation of the whole retina. Purpura, Tranchina, Kaplin & Shapley [48] measured TCSFs in magnocellular and parvocellular primate ganglion cells at up to five adaptation levels. They modelled the TCSF data using a linear systems model with—as in our model—two lead-lag filters but they added an implausibly large and variable number of sometimes extremely fast LP-filters. In general, their data are consistent with the psychophysical measurements described here, except as they noted (p.89) the ganglion cell TCSFs remained bandpass even at low adaptation levels. This agrees with our model in which *k*, the feedforward gain, when restricted to conditions that are cone-mediated, is constant. As well as showing a speeding up of the visual response, their data also show that adaption begins at backgrounds as low as 1 log_10_ trolands.

Lee, Pokorny, Smith, Martin & Valberg [77] measured chromatic and luminance TCSFs in primate magnocellular and parvocellular ganglion cells and in human observers. The magnocellular and human luminance TCSFs were comparable in form, but unsurprisingly the human data showed lower temporal acuities, a difference that Lee *at al*. attributed to the interposition of a later 4-stage LP-filter with a 20-Hz corner frequency. In our model, the late filters are two LP-stages with fixed 30-Hz corner frequencies. Other important features of their results are that adaptation becomes apparent between 0.3 and 1.3 log_10_ trolands, consistent with Purpura *et al*., and that the TCSFs and the phase delays measured together show that a linear systems approach to modelling such data is appropriate.

Lastly, Dunn, Lankheet & Rieke [68] measured not only cone and bipolar cell responses in the same preparation, but also midget and parasol ganglion cell responses. They found that adaptation switched from postreceptoral to receptoral as the light level increased. Between darkness and 1000 R*/s (photoisomerizations per second) they found evidence for frequency-dependent adaptation in the ganglion cells, but not in either the bipolar cells or cones, and above 1000 R*/s they found adaptation mainly in cones (see Figure 3 in [68]). To incorporate this postreceptoral adapting stage in our model, we have placed one of the variable-speed LP-stages after the feedforward stages. Psychophysical evidence for postreceptoral adapting stages similar to those in our model comes from the work of Stockman, Candler & Sharpe [78], who measured rod TCSFs and rod phase delays from -3.3 to 0.8 log_10_ scotopic td and modelled the changes in both by changing the corner frequencies of LP-stages. Since over most of this range, the rate of photon absorption per rod is too low for sensitivity regulation to be practicable within the rod photoreceptor itself, the regulation must occur postreceptorally (see [78]). We speculate that one of these stages might correspond to adaptation in the ganglion cells, which the rod and cone pathways have in common (see, for example, Figure 1 in [79]).

In conclusion, based on a review of physiological data we have organised the adaptation stages of the model as illustrated in Fig 8.

As in most other models, noise seems to play little or no role in altering the shapes of the TCSFs, which can be accounted for deterministically by the model. Thus, we implicitly assume that temporal sensitivity does not critically depend on early sources of noise; *e*.*g*., quantal fluctuations. This is consistent with previous studies that examined the role of noise on flicker detection, and which suggested early noise is not a limiting factor in human cone-mediated vision [18, 80].

### Cone independent and non-independent adaptation

The locations of the adaptation elements within the visual pathway have clear implications as to whether the adaptation is likely to be cone-specific, often referred to as first-site adaptation, or non-cone-specific, often referred to as second-site adaptation (discussed, for example, in [81, 82]). In the model, we have assumed that the initial frequency-independent gain control, *g*, and the first three variable-speed LP-filters are likely to be within the photoreceptor. Given this, these elements represent sites of first-site, cone-specific adaptation. The two inhibitory subtractive feed-forward stages in the model, in contrast, are assumed to be after the photoreceptors, and are thus sites of second-site adaptation. Since the feedforward is inhibitory, these second-sites can be cone-opponent under some conditions. The fourth variable-speed LP-filter is also assumed to be postreceptoral and so is a site of second-site adaptation site. At this site, signals from other cones (and even rods) may speed up the common filter. We speculate that the shapes of the Stiles’ π_4_ and π_5_ field spectral sensitivities [83] may reflect background adaptation mediated at such a second-site where the sensitivity of π_4_ (M-cone-detected) or of π_5_ (L-cone-detected) lights can be raised by the other cone type. Thus, the field spectral sensitivity of π_4_ is higher than the isolated M-cone spectral sensitivity at long-wavelengths because L-cones raise M-cone detection sensitivity at that second-site, while the field spectral sensitivity of π_5_ is higher than the isolated L-cone spectral sensitivity at short-wavelengths because the M-cones raise L-cone detection sensitivity at short-wavelengths.

The current rapid progress in retinal physiology may soon allow some of these details and ambiguities to be clarified.

### Psychophysical implications of the model

**Fig 11:**
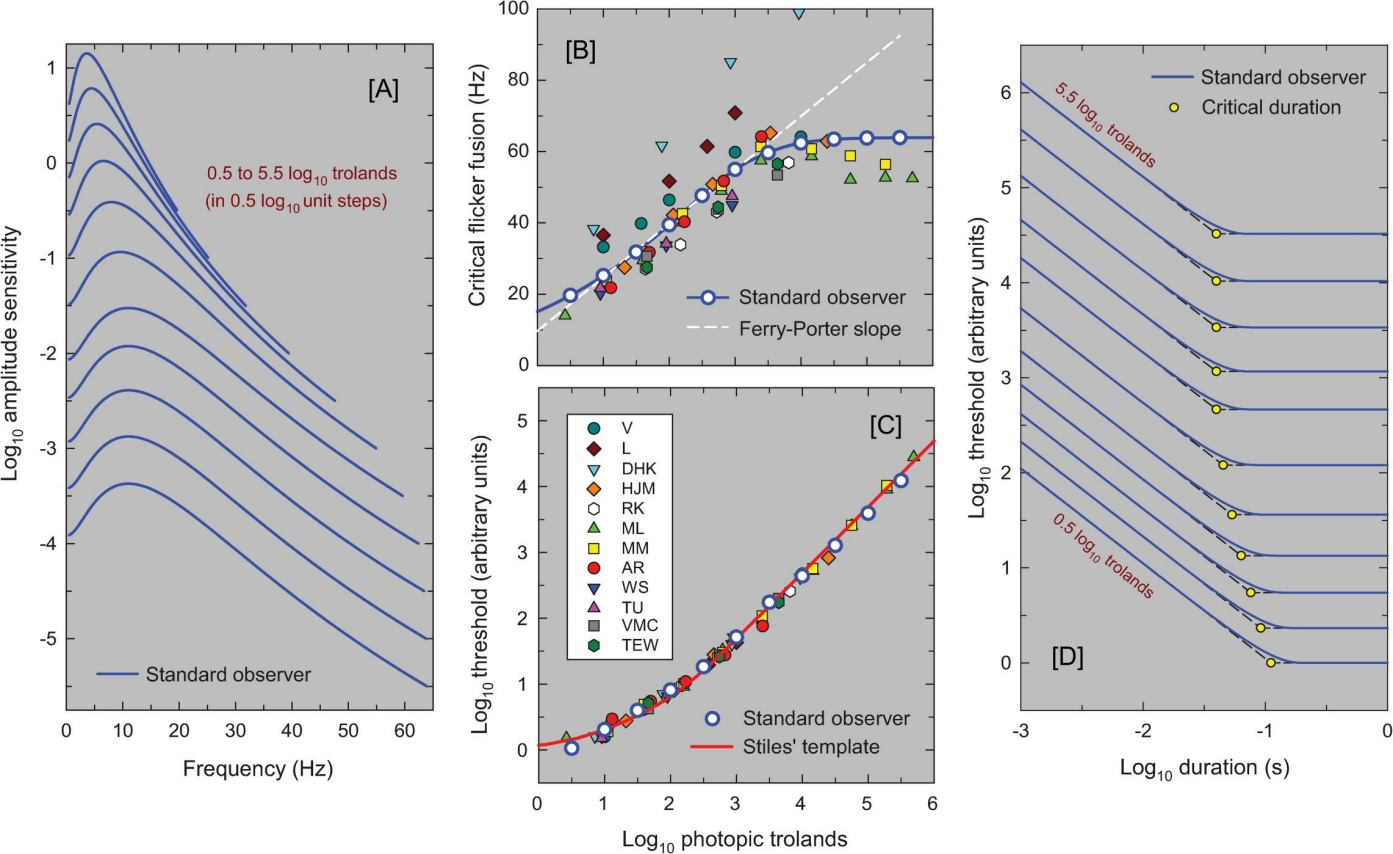
The panels show the standard model predictions. Panel [A] shows the predicted TCSFs as amplitude sensitivities at a range of light levels (0.5 to 5.5 log_10_ trolands in 0.5 log_10_ steps). The TCSFs end at the assumed CFF for each level. Panel [B] shows the predicted CFFs extrapolated from the individual TCSF fits (as coloured symbols) and the predictions of the standard model shown as the continuous blue line, the white circles are in 0.5 log_10_ unit intervals and correspond to the line endings in Panel [A]. The dashed white line is the best fitting Ferry-Porter slope to the standard model. Panel [C] shows predicted threshold-versus-intensity (TVI) curves for brief flashes. The red line is Stiles’ template for TVI curves. The colored symbols for individual observers have been vertically shifted to align with Stiles’ template removing individual differences in overall gain. Panel [D] shows the predicted flash thresholds as a function of flash duration (both on log scale) for a range of light levels from 0.5 to 5.5 log_10_ trolands in 0.5 log_10_ steps. We have added dashed straight lines to indicate complete temporal summation at short durations (with slopes of -1) and the long duration asymptote (with slopes of 0). The duration at which these lines intersect is the “critical duration” indicated by the yellow circles.

Panel [A] of Fig 11 shows the TCSF predictions for the standard observer plotted at background steps of 0.5 log_10_ trolands from 0.5 to 5.5 log_10_ trolands. These can be compared with the individual TCSFs plotted in Figs 2–6, above. The new model provides fundamental insights into the workings of the visual system and can be used to model other measures using periodic and aperiodic stimuli. For example, each TCSF shown in Panel [A] ends at the frequency at which the amplitude threshold for the standard observer reaches the maximum 100% contrast for sinusoidal flicker (at which the amplitude sensitivity is 1/*I*, where *I* is the background luminance). This corresponds to the temporal acuity limit or critical flicker (or fusion) frequency (CFF) for that level. The CFFs from Panel [A] are shown as the white circles plotted as a function of log_10_ luminance in Panel [B]. The solid blue line shows the continuous function.

One important characteristic that is seen when the CFF is plotted against the logarithm of light level, is that over much of the range the function follows a straight line, a behavior referred to as obedience to the Ferry-Porter law [8, 9]. The dashed white line in Panel [B] shows the straight line that best fits the standard observer between 1 and 3 log_10_ trolands. It has a slope of 15 Hz per decade. The model obeys the Ferry-Porter law over at least a 2 log_10_ unit range, even though in deriving the model, no attempt was made to produce this result. Shown as symbols in Panel [B] are the CFFs calculated using the model and best-fitting parameters for each observer. The individual CFF predictions are also consistent with the Ferry-Porter law.

Panels [C] and [D] of Fig 11 show the model predictions for aperiodic stimuli. Although the model was developed to explain data in the frequency domain, its time domain representation, which is given in Equation (B) in S1 Appendix, can be used to predict the visual response to any stimuli. For example, we can calculate the response to brief flashes over a range of mean light levels. In order to extract a measure of visual sensitivity from these responses, we follow Roufs [46] in assuming that a flash will be detected if the peak response exceeds some critical threshold that does not depend on the mean light level. Flash sensitivity will then be proportional to the peak of the impulse response, or equivalently flash threshold will be inversely proportional to the peak response. While Roufs assumed that the same threshold applies to flickering and flashed stimuli, we do not. For example, an above-threshold flickering light at 10 Hz will exceed this hypothetical threshold 10 times every second (or 20 if increments and decrements are detectible and have the same threshold), whereas an above-threshold flash will exceed it only once, so any probability summation [28] may reduce the flicker detection threshold relative to the flash threshold. Accordingly, the units of our implied thresholds are somewhat arbitrary as they depend on the unknown strength and duration of probability summation for each observer.

**Fig 12:**
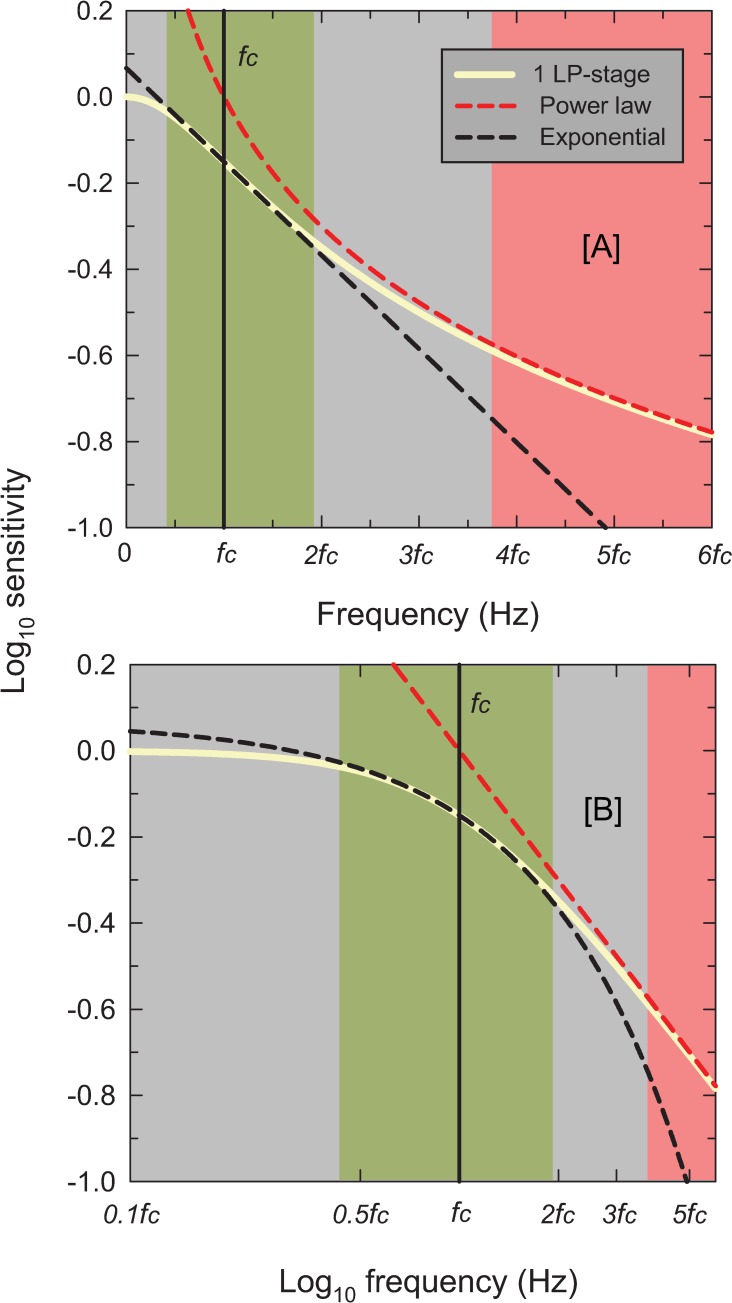
The solid cream-colored lines show the logarithmic relative amplitude response of one LP-stage plotted as a function of frequency on a linear scale in Panel [A] and on a logarithmic scale in Panel [B]. The maximum response has been normalized to 1. The corner frequency, *f*_*c*_, is indicated by the vertical black lines, and frequency is marked off in multiples of *f*_*c*_. The red dashed lines show the asymptotic frequency response, which in panel [B] is a straight line with a slope of -1 consistent with there being just one stage. The red region highlights the range above 3.74 *f*_*c*_ where the filter response is within 0.015 log_10_ unit of the asymptotic response. The black-dashed lines, indicating exponential loss of sensitivity with increasing frequency and which describe the measurements shown in Figs 2–6, are the best exponential fit to the single LP-filter (cream lines) between 0.33*f*_*c*_ and 2.0*f*_*c*_. The green region highlights the range from 0.43 to 1.92 *f*_*c*_ within which the filter is within ±0.015 log_10_ unit of the exponential response (which is a straight line in Panel [A]).

Substituting the parameters from the individual fits to the observed TCSFs into the impulse response function of Equation (B) in S1 Appendix, we reconstructed flash responses for different mean luminances and extracted the height of the peak. These have been plotted as threshold versus intensity (TVI) functions on a double logarithmic scale in Panel [C] of Fig 12 (coloured symbols). Note that thresholds for each observer were normalised (vertically aligned) to account for potential differences between the flicker and flash sensitivities. We also used the standard model functions to estimate flash thresholds as a function of mean luminance between 0.5 and 5.5 log_10_ trolands in 0.5 log_10_ unit steps (white circles in Panel [C]). We have also plotted as the solid red line Stiles’ TVI template (from Table A in [83]) on the same scale, and the agreement is remarkably good. The model also captures two well-known psychophysical laws: first, the threshold increases in proportion to the background at high light levels (Weber’s law), and second, at low levels threshold increases roughly as the square-root of the background (the de Vries-Rose law). The model suggests that Weber’s law is followed at high light levels largely due to bleaching and other frequency-independent gain changes, while the de Vries-Rose law occurs because of increasing temporal summation at progressively lower levels. Note, neither quantal fluctuations nor square-root gain changes, both of which have been invoked to explain the de Vries-Rose law, have any place in our model.

Additionally, we can use the derived impulse responses to predict the peak response to flashes of different durations on backgrounds of different intensities. Measured thresholds decrease in proportion to flash duration for short durations (temporal summation, also known as Bloch’s law) but thresholds plateau at a constant value for long durations [17, 84]. The flash duration at which thresholds transition from Bloch’s law to a constant value is known as the critical duration and has been shown to decrease with increased luminance. The predictions of our model for flash durations of 1 ms to 1 s and luminances of 0.5 to 5.5 log_10_ trolands are shown in Panel [D] of Fig 11. These curves have all been normalised relative to the smallest amplitude threshold (for long flashes on dim backgrounds). The predictions can be seen to agree with Bloch’s law at short durations and plateau at longer durations. Thresholds increase with increasing luminance. The critical duration increases from about 110 ms in dim light to about 40 ms in bright light (yellow circles).

### High-frequency exponential loss of sensitivity

The next issue we address is how a system made up of our LP-stages can produce an exponential sensitivity loss at high-frequencies even though the expected asymptotic loss at high-frequencies for a cascade of *n* LP-stages is $f^{-n}$ (i.e., not exponential but a power law and having a slope of *-n* on double logarithmic co-ordinates).

Panel [A] of Fig 12 shows the logarithm of the amplitude response of one LP-stage—a single leaky integrator (cream solid line)—as a function of frequency (linear scale). The single stage has a corner frequency of *f*_*c*_ marked by the vertical black line. Panel [B] shows the same response on a logarithmic frequency axis. In both cases frequency is indicated in multiples of *f*_*c*_. Above about 3.74*f*_*c*_ (where *f* >> *f*_*c*_) the response of a single LP-stage in the log-log coordinates of Panel [B] approaches an asymptotic line (red dashed line) with a slope of -1 determined by a power law with exponent 1—the value of *n* in the case of a single LP stage. That line in the log-linear coordinates of Panel[A], produces a function that becomes shallower with increasing frequency. The red shaded regions in Panels [A] and [B] show where the power-law approximation is a good fit to the LP response. How can a system made up of LP-stages, then, have the exponential high-frequency characteristics of the data in Figs 2–6, which are linear in log-linear coordinates of Panel [A] and follow the dashed black line? The explanation is simply that the response of a single LP-stage *approximates* an exponential decay (and thus a straight line in Panel [A]) over the range of frequencies restricted to lie between about 0.36*f*_*c*_ and 1.92*f*_*c*_, as shown by the green-shaded regions. This argument applies equally to cascades of multiple LP-stages but produces a steeper slope. (A mathematical explanation involving a Taylor’s expansion around *f*_*c*_ is provided in S1 Appendix.) The introduction of subtractive inhibition shifts the range of approximately exponential loss to slightly higher frequencies and slightly reduces the exponential slope of the decline, see S1 Appendix.

In conclusion, our analysis shows that the response of systems of LP-stages approximates an exponential function over much of the mid-frequency range. At higher frequencies, as indicated by the red regions in Fig 12, responses would more closely approximate a power law, but these frequencies are above the flicker fusion limit and are therefore invisible—flicker cannot be seen, and flicker sensitivity cannot be directly measured.

The exponential decline in sensitivity with frequency demonstrated by our model is consistent with the empirical data, but it is not a feature that is unique to our model. Any model built from cascades of several LP-stages produces approximately exponential functions over frequency ranges that depend on the number of stages and their speeds. Another, and mathematically simpler, way of ensuring an exponential decline would be fit a frequency response that truly is, or tends towards, an exponential function at high frequencies [24, 38, 85], but we are unaware of any physically-plausible motivation for such a model. Indeed, Kelly has pointed out that a strict adherence to an exponential frequency response would produce a non-causal impulse response [86]. One other significant, physically motivated, class of models that has been proposed to account for TCSF data is based on solutions to diffusion equations [43, 86, 87]. Predictions of these models, when log_10_ sensitivity is plotted against linear frequency, do not produce straight lines but also have a large negative acceleration, which is inconsistent with most TCSF data.

### Relation between corner frequency and the high-frequency slope

**Fig 13:**
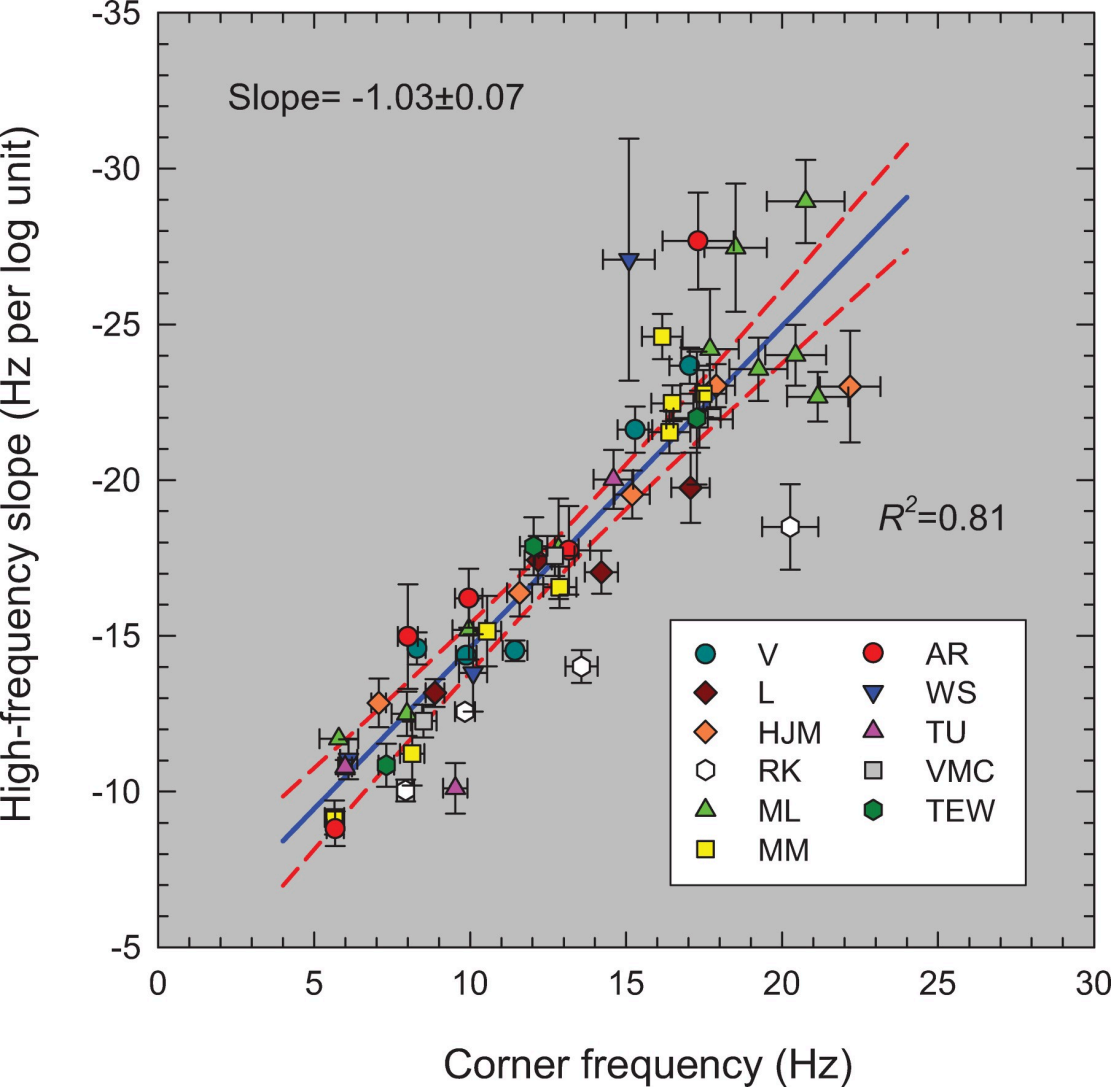
Best-fitting slopes (Hz per log_10_ unit sensitivity) for the linear high-frequency sensitivity losses from table A as a function of the corresponding best-fitting corner frequencies (Hz) from table B for the amplitude-sensitivity data shown in Figs 2–6. (Note that the slope is plotted to become more negative upwards.) The solid blue line is the linear regression line, which has a slope of -1.03±0.07, an intercept of -4.28±0.97 Hz per log_10_ unit, and an adjusted *R*^*2*^ of 0.81. The dashed red lines are the 95% confidence intervals for the regression. Only the parameters for levels thought to be cone-mediated are shown.

For each TCSF, we carried out two fits. First, we found the slopes, given in Table A, that best fit the exponentially falling high-frequency sensitivities at each light level. Second, we applied the new model and found the best-fitting corner frequencies and overall gains given in Table B in S1 Appendix. In this section, we consider the relation between the high-frequency slopes and the corner frequencies for those mean intensity levels that can be assumed to be cone-mediated. Fig 13 shows the best-fitting high-frequency slopes (in Hz per log_10_ unit of sensitivity) against the best-fitting corner frequency (Hz). The error bars show ±1 standard error of the fitted parameter. The discrepant values for DHK were not included. The blue line shows the linear regression and the 95% confidence interval of the fit lies between the dashed red lines. The best fitting line has a slope of -1.03±0.07.

It is immediately obvious in Fig 13 that the exponential high-frequency slopes and corner frequencies are almost exactly linearly related. This simple relation means that a quick and efficient estimate of the speed of the visual system at any mean retinal illuminance can be determined from the high-frequency slope of the TCSF by measuring two or three contrast thresholds (at mid to high frequencies) and using Fig 13 to estimate the corresponding corner frequency and hence the time constant τ. Such an abbreviated method is likely to be especially useful in clinical or screening contexts where time is limited.

## Summary

We propose a simple model of human light adaptation that combines a frequency-independent gain control, a cascade of LP-filters and two stages of subtractive inhibition with just two intensity-dependent parameters: one that controls together the corner frequencies (or time constants) of six of the eight LP-filters, and a second that controls the frequency-independent gain at higher light levels. The model accounts for TCSF data collected over the past 65 years, including low-frequency attenuation, the exponential fall in high-frequency sensitivity, and the adaptation-dependent sensitivity changes at both low and high frequencies.

The model is agnostic as to the location and sequence of its elements in the visual pathway. However, following a review of the available physiological data we have tentatively placed the model elements at specific locations within the pathway.

As we show, the model can be easily extended to aperiodic as well periodic (flickering) stimuli. Our next goal will be to test and develop the model by applying it to new periodic and aperiodic data collected in the same observers.

Details of the model can be easily modified to accommodate new psychophysical and physiological data as they become available.

## Supporting information

S1 Appendix Justification for data excluded because of rod intrusion. Time-domain representation of the model. Analysis of exponential frequency response. Why leaky integrators might have similar corner frequencies. Effect of subtractive inhibition on exponential frequency response. High-frequency linearity and low frequency Weber’s law. Also, Table A: Best-fitting high-frequency exponential slopes, and Table B: Best-fitting model parameters.
(PDF)

S1 Dataset Historical datasets plotted in Figs 2–6 and used to develop the model. (XLSX)
