# Supplementary material for: Light adaptation controls visual sensitivity by adjusting the speed and gain of the response to light
Source: PLoS One. 2019 Aug 7;14(8):e0220358. doi: 10.1371/journal.pone.0220358 (PMC6685682; doi:10.1371/journal.pone.0220358)
Supplement: S1 Appendix — Time-domain representation of the model. Analysis of exponential frequency response. Why leaky integrators might have similar corner frequencies. Effect of subtractive inhibition on exponential frequency response. High-frequency linearity and low frequency Weber’s law. Also, Table A: Best-fitting high-frequency exponential slopes, and Table B: Best-fitting model parameters. (PDF) [file pone.0220358.s001.pdf]

Supporting information

Rod intrusion

We excluded TCSF data from the model development that was likely to be influenced by rod-mediated detection of the flicker. Except for the TCSF experiments of Stockman *et al.* [1] shown in Fig 5, in which rod-mediated detection was precluded, little attempt was made to exclude rod involvement at lower light levels in most TCSF studies. Some rod intrusion could be expected at the lower levels in the experiments of De Lange [2], Kelly [3], Roufs [4] and Rovamo *et al.* [5], who used circular 2°, 68°, 1°, and 1.66° centrally-viewed white flickering targets, respectively. With good fixation control, the small centrally-viewed targets used by all but Kelly might be expected to fall mainly in the rod-free fovea and thus produce mainly cone-detected flicker. However, the need for Roufs to dark-adapt his subjects for up to 45 minutes at low light levels (see p. 263 of [4]) and De Lange for 15 minutes [“with the (room) light switched off” at lower levels (see p. 49 of [6]) suggests that rod detection was important and central fixation was not necessarily well maintained. Rod intrusion should be less for Swanson *et al.* [7], who used a light that was metameric with 600 nm, and least for von Wiegand *et al.* [8], who used a 635-nm target light, but in both cases some rod intrusion is possible at low temporal frequencies and low luminances (e.g. [9, 10]).

Since there is likely to be rod intrusion in the data at low levels in Figs 2-4 and 6, we were faced with the problem of determining which levels should be excluded from our analysis so that we could be confident that we were modelling cone-mediated vision.

Broadly speaking, rod saturation begins at about 100 scotopic trolands [11-13], which corresponds, for example, to about 40 to 70 *photopic* trolands for standard whites such as Illuminants D65 and A (Table 1(2.4.4) of [14]). Thus, a conservative cut-off might be about 40 photopic trolands. However, based on the form of the data and the fact that the experimental conditions for De Lange and Roufs did not favour rod-detection, we have chosen a lower cut-off near 10 photopic trolands. Additionally, this cut-off coincides with the level below which the parameter *k* in the model*,* when allowed to vary*,* began to decrease and the TCSF to change in shape from band-pass to low-pass. Consequently, a plausible simplification, and an assumption that we adopted in our modelling, is that *k* is constant for cone-mediated vision.

Time-domain representation of the model

Going from the frequency domain to the time domain strictly requires knowledge of the phase response as well as amplitude response (Equation (2)) of the system. However, we note that the phase responses of LP-stages are determined by their temporal response (which is an exponential decay over time), and our model can be represented in the complex Fourier domain as,

$$H(f)=g\frac{\left(if+(1-k)f_c\right)^2}{\left(if+f_c\right)^6\left(if+f_{cL}\right)^2}\ , \qquad \text{(A)}$$

where $i=\sqrt{-1}$. The triphasic temporal response, *R(t)*, of the model is found by taking the inverse Fourier transform of Equation (A), which gives in terms of time constants, i.e., $\tau=\frac{1}{2\pi f_c}$ and $\tau_L=\frac{1}{2\pi f_{cL}}$:

$$y(t)=g\left(Ae^{-\frac{t}{\tau}}+Be^{-\frac{t}{\tau_L}}\right), \qquad \text{(B)}$$

where:

$$A(t)=\sum_{u=0}^{5}\frac{\left(\frac{k}{\tau}\right)^2\frac{(6-u)}{u!}(Xt)^u}{X^7}+\sum_{v=0}^{4}\frac{2\frac{k}{\tau}\frac{(5-v)}{v!}(Xt)^v}{X^6}+\sum_{w=0}^{3}\frac{\frac{(4-w)}{w!}(Xt)^w}{X^5}$$

$$B(t)=\sum_{z=0}^{1}\left\{\frac{\left(\frac{k}{\tau}\right)^2(Xt-6)}{X^7}+\frac{2\frac{k}{\tau}(Xt-5)}{X^6}+\frac{(Xt-4)}{X^5}\right\}.$$

and,

$$X=\frac{1}{\tau}-\frac{1}{\tau_L}$$

Equation (B) has been plotted in the lower right orange panel of Fig 8 with *k*=0.80, f_c=15 and f_{cL}=30; *g* has been chosen to normalise the peak to 1.

The amplitude response of a cascade of leaky integrators is asymptotically a power law, but exponential for a range of visually significant frequencies

The amplitude response, *A(f)*, of a single stage of leaky integration (low-pass filtering), or an "LP-stage" for short, is given by,

$$A(f)=\frac{c}{\sqrt{f^2+f_c^2}} \quad \text{or} \quad \log_{10}\left(A(f)\right)=C-\frac{1}{2}\log_{10}\left(f^2+f_c^2\right), \qquad \text{(C)}$$

where f is frequency in Hz, $C=\log_{10}(c)$ is the logarithm of the overall gain, $f_c=1/2\pi\tau$ is the corner (or cut-off) frequency of the low-pass filter where τ (seconds) is the time constant (or integration time) of the leaky integrator. This logarithmic form of Equation (C) has been plotted against frequency in Panels [A] and [B] of Fig 12 as the solid cream lines. The high frequency asymptote, when f is large compared to f_c, is given by,

$$A(f)\approx cf^{-1} \quad \text{or} \quad \log_{10}\left(A(f)\right)\approx C-\log_{10}(f), \qquad \text{(D)}$$

which is a power law function of f, with an exponent of minus one. Plotted on double logarithmic co-ordinates (see the dashed red line in Panel [B] of Fig 12) this becomes a straight line with a slope of minus one. However, this approximation is valid only when the frequency is high, say above 3 times the corner frequency, as shown by the red shaded region in Fig 12. At lower frequencies the amplitude response is much better approximated by an exponential function, which is a straight line when log amplitude is plotted on a linear frequency scale (Panel [A]). To understand why, consider the Taylor series expansion of the logarithmic response function (Equation (C)) around $f=f_c$. (We choose to expand around f_c as it is an inflection point of $\log_{10}(A)$, i.e., the second derivative is equal to zero at this point and there is no second-order term in the Taylor expansion and so $\log_{10}(A)$ will be closest to a straight line at this point.)

$$\begin{aligned}\log_{10}\left(A(f)\right)&=C-\frac{1}{2}\log_{10}\left(f^2+f_c^2\right)\\ &=C-\frac{1}{2\log_e(10)}\left[\log_e\left(2f_c^2\right)-\frac{1}{f_c}(f-f_c)+\frac{1}{6f_c^3}(f-f_c)^3-\frac{1}{8f_c^4}(f-f_c)^4+\mathrm{O}(f-f_c)^5\right].\end{aligned} \qquad \text{(E)}$$

For values of f close to f_c the expansion will be dominated by the constant and linear terms, i.e.,

$$\log_{10}\left(A(f)\right) \approx D - \frac{1}{2\log_e(10) f_c}(f - f_c), \quad \text{(F)}$$

where $D = C - \frac{\log_e\left(2f_c^2\right)}{2\log_e(10)} = \log_{10}\left(A(f_c)\right)$. This function has been plotted in both panels of Fig 12 as the dashed black line and can be seen to be a good approximation to the filter shape between about $0.36f_c$ and $1.92f_c$ (i.e., the error is between ±3.5% in the green shaded region), over which sensitivity declines by about 0.31 $\log_{10}$ units (i.e., by about 51%).

For a cascade of LP-stages in a linear system the convolution theorem states that the total response of the cascade is equal to the product of the responses of the individual stages. Or, equivalently, the logarithm of the cascade response is the sum of the logarithms of the individual responses. At high frequencies *n* LP-stages will tend toward a power law with an exponent of minus *n* (a straight line of slope minus *n* when plotted on double logarithmic coordinates). This result has been noted many times before [15, 16] and has occasionally been used as justification for rejecting a leaky integrator model altogether [17] because the *measurements* at high frequencies don't assume this form. However, between about $0.36f_c$ and $1.92f_c$ a cascade of *n* identical LP-stages has an approximately exponential frequency response with an exponent of $-n/\left[2\log_e(10) f_c\right]$, and sensitivity will decline by about $0.31n$ $\log_{10}$ units. A cascade of six such stages will have an approximately exponential response over 1.86 $\log_{10}$ units, close to the maximum flicker sensitivity range seen in TCSFs. This range crucially depends on the LP-stages having the same corner frequencies, if the corner frequencies differ the frequency ranges over which they each approximate an exponential function will only partially overlap and the good exponential approximation to the TCSF will cover a smaller region. The value of the exponent of the approximation in this region, i.e., the slope of the linear region when log sensitivity is plotted against linear frequency, will be $-\frac{1}{2\log_e(10)}\sum_{j=1}^{n}\frac{1}{f_{cj}}$ where f_{cj} denotes the corner frequency of the jth LP-stage.

For a cascade of leaky integrators all the corner frequencies (or equivalently, all the time constants) should take similar values.

In addition to producing the largest range of frequencies over with the TCSF is approximately exponential, it is also the case that having identical corner frequencies produces maximal temporal

contrast sensitivity at all frequencies. Consider the simplest case with two stages with corner frequencies C_1 and C_2. The amplitude response, *A(f)*, is then given by:

$$A(f)=\frac{C_1C_2}{\sqrt{f^2+C_1^{\,2}}\sqrt{f^2+C_2^{\,2}}}, \qquad \text{(G)}$$

where *f* is temporal frequency in Hz. Note that the numerator is the product of the corner frequencies in order to set the low frequency asymptote to 1. The high-frequency asymptote on a log-log plot is then a straight line with slope -2 which passes through the point (C_1C_2, 1). The way we have defined our amplitude response means the low-frequency limb is fixed at *A*=1 for all C_1 and C_2 and the location of the high-frequency limb depends solely on the product of C_1 and C_2, so the family of curves that have the same low- and high-frequency limbs can be parametrised as $C_1=C/X$ and $C_2=CX$, for some *C* (which is the geometric mean of C_1 and C_2). C and *X* are both greater than 0. In the case when the corner frequencies are identical, i.e., *X*=1, Equation (G) reduces to:

$$A_s(f)=\frac{C^2}{(f^2+C^2)}, \qquad \text{(H)}$$

while if $X\neq 1$:

$$A_d(f)=\frac{C^2}{\sqrt{f^2+\left(\frac{C}{X}\right)^2}\sqrt{f^2+(CX)^2}}, \qquad \text{(I)}$$

where the subscripts *s* and *d* denote corner frequencies that are the *same* or *different*. The question is which of these two amplitude responses is larger for any given frequency. We note that *C*, *X* and *f* are all strictly positive and so both A_s and A_d are positive, which therefore means that $A_s>A_d$ if, and only if, $A_s^{\,2}>A_d^{\,2}$. We have:

$$A_s^{\,2}=\frac{C^4}{\left(f^4+2C^2+C^4\right)} \text{ and } A_d^{\,2}=\frac{C^4}{f^4+\left(\frac{1}{X^2}+X^2\right)+C^2+C^4}, \qquad \text{(J)}$$

so that (by inspection of the denominators in Equations (J)), $A_s^{\,2}>A_d^{\,2}$ if, and only if, $2<\frac{1}{X^2}+X^2$ and this is clearly true for all $X\neq 1$ and is independent of *f*. So, we see that having stages with identical corner frequencies will always lead to optimal sensitivity at all frequencies compared to two stages

with different corner frequencies. The above reasoning can be easily extended to the case of more than two filters.

A more intuitive explanation of why having identical corner frequencies is optimal is to consider a serial cascade of two filters (leaky integrators), one of which is slower (has a lower corner frequency) than the other. If the slower filter comes first, then the second filter will be capable of passing higher frequencies than it receives as input and its sensitivity would be wasted. Similarly, if the slower filter comes second then the first filter passes high frequencies which the second attenuates, which again would be wasteful. If the filters have the same corner frequency, then they are optimally tuned to pass (or attenuate) the same set of frequencies. While it is highly unlikely that all the significant LP-stages of light adaptation in the visual system have identical time constants, any process which seeks to optimise the efficiency of the system might be expected to make them as near to identical as possible.

Subtractive inhibition maintains the exponential fall-off but reduces its slope and shifts it to higher frequencies

While a cascade of LP-stages can produce an exponential decline in sensitivity over the visible range of flicker frequencies, it does not explain the loss of sensitivity at low frequencies. We model the loss of low-frequency sensitivity as the result of inhibition. A standard high-pass filter can be constructed by passing a signal through a leaky integrator with unity DC gain and subtracting the result from the original signal. If the DC gain is less than unity, the filter will only partially cancel signals at low frequencies. These kinds of filter can be thought of as partial high-pass filters and are often used in electric engineering (where they are referred to as “lead-compensators”) in order to increase stability in control circuits and sharpen the temporal response. The amplitude response of such a filter is given by:

$$A(f)=\frac{\sqrt{f^2+\left((1-k)f_c\right)^2}}{\sqrt{f^2+f_c^2}}\ , \qquad \text{(K)}$$

where k is the gain of the filter whose response is to be subtracted and can be thought of as the strength of inhibition. Note that when k=1 there is complete inhibition at low frequencies; i.e., a standard high-pass filter, while for k=0 there is no inhibition and the numerator and denominator in Equation (K) cancel, *i.e.*, an all-pass filter. Our model is made up of 6 LP-stages and two lead-

compensators with the same DC gain. Based on previous work we assume that two central LP-stages do not light adapt while the other four LP-stages do. This is also consistent with the idea that a major limiting factor of visual sensitivity is the dynamic range of post-receptoral spiking neurons, so the processes of light adaptation should be most pronounced early in the visual pathway. For simplicity, and to account for the large frequency range of exponential approximation, we assume that the variable LP-stages change together and the filters in the inhibition adapt in the same way. The equation for this model is given by,

$$A(f) = g\frac{f^2 + \left((1-k)f_c\right)^2}{\left(f^2 + f_c^{\,2}\right)^3 \left(f^2 + f_{cL}^{\,2}\right)} \quad \text{or}$$

$$\log_{10}\left(A(f)\right) = G + \log_{10}\left(f^2 + \left((1-k)f_c\right)^2\right) - 3\log_{10}\left(f^2 + f_c^{\,2}\right) - \log_{10}\left(f^2 + f_{cL}^{\,2}\right), \qquad \text{(L)}$$

where f_c and f_{cL} are the corner frequencies of the variable and fixed stages, respectively. This function, where $G=\log_{10}(g)$, was fitted to the TCSF data by varying G, f_c, f_{cL} and k using a nonlinear least squares procedure in SigmaPlot or MATLAB. Note that f_{cL} was fixed across observers and light levels, k was fixed separately for each observer (except at low levels), and G and f_c varied both with observer and with light level. The fits are plotted as the black or gray lines in Figs 2-6. The shape of Equation (L) depends on k, f_c and f_{cL}.

The general equation for our full model does not lend itself to a simple Taylor series expansion as it did in the single leaky integrator case, so instead we use a technique from computer vision to examine the approximate straight-line portion of the log sensitivity versus linear frequency plot, namely the Hough transform [18]. Any straight-line can be parameterised by two quantities, one denoting a direction and one denoting a position, often given as the slope and the intercept of the y-axis. In the Hough transform these quantities are typically taken as the orientation of the line, ϑ, and its shortest distance to the centre of the image, ρ. In computer vision, the transform is used to map each point in an image to a segment of a sinusoidal curve through ϑ-ρ space. Points that are collinear will produce curves that intersect at a single point (ϑ, ρ); i.e., a straight-line in the image will appear as a peak in the summed distribution of curves in ϑ-ρ space. We generated an image of the TCSF curve (with values of f_c=15, f_{cL}=30 and k=0.8) and applied the Hough transform to extract the largest (approximately) straight line in the image. From this we can find the range of the linear fit in terms of sensitivity (~1.75 $\log_{10}$ units) and frequency (in this case, between about 13.1 and 47.1 Hz), and the slope is -19.4 Hz/decade.

High-frequency linearity and low frequency Weber's law

At high frequencies (when $f >> f_c$ & f_{cL}) the equation for our model (J) simplifies to:

$$A(f) \approx \frac{g}{f^6}, \tag{M}$$

which, in the traditional double logarithmic coordinates of Bode [19], is a straight-line with slope of -6 passing through $log_{10}(g)$ at 1 Hz where $-6\ \log_{10}(f) = 0$. If the high-frequency power law region of each TCSF were within the visible range, then high-frequency linearity would hold if and only if g were constant. Inspection of Panel [B] of Fig 9 shows this is approximately true up to about 3.5 $\log_{10}$ Tds. However, as noted above, the region where the power law approximation holds lies largely above the temporal acuity limit above which sensitivity cannot be measured and below which TCSFs are approximately exponential functions. In semi-logarithmic coordinates the exponential losses in sensitivity appear as straight-lines of different slopes, and so cannot strictly conform to "high-frequency linearity", i.e., they could only coincide at a single intersection point, not over an extended range. Plotted on a logarithmic frequency scale, however, these lines accelerate downwards and only appear to coincide. We suggest that the notion of high-frequency linearity is an inappropriate inference based on the way in which amplitude sensitivity has been plotted in the past; it is not a feature of visual sensitivity.

At low frequencies (when $f << f_c$ & f_{cL}) our model (J) simplifies to:

$$A(0) = \frac{g(1-k)^2}{f_c^{\,4} f_{cL}^{\,2}} \tag{N}$$

Now Weber's Law holds if $\Delta I/I$ is constant, where $\Delta I = A$ is the amplitude threshold. In our model k and f_{cL} are fixed across light levels, but g and f_c vary, and, from Equation (N), Weber's law will hold if:

$$\frac{f_c^{\,4}}{Ig} = W, \tag{O}$$

where W is a constant that is proportional to the Weber fraction. Below about 3.4 $\log_{10}$ Td, g is constant (Fig 9[B]) so Weber's law will hold if f_c increases in proportion to the 4th root of I. However, the best fitting power law function relating f_c to I (see Equation (3), plotted as the blue curve in Fig 9[A]) has an exponent of 0.181, which corresponds to the 5.5^{th} root of I. Above 3.4 $\log_{10}$ Td, f_c is constant while g decreases in proportion to I (see Equation (4) and Fig 9[B]), so here W will be constant. Thus, according to our model, Weber's law is not strictly maintained at low frequencies at

low- to mid-light levels. Note that low-frequency flicker thresholds are distinct from flash thresholds, which do indeed show good adherence to Weber's law over a large range of light levels [20], but this will likely depend on the shape and size of the impulse response rather than flicker sensitivity at any specific frequency.

References

1. Stockman A, Langendörfer M, Smithson HE, Sharpe LT. Human cone light adaptation: from behavioral measurements to molecular mechanisms. J Vision. 2006;6(11):1194-213. doi: 10.1167/6.11.5. PubMed PMID: 17209729.
2. de Lange H. Research into the dynamic nature of the human fovea-cortex systems with intermittent and modulated light. I. Attenuation characteristics with white and colored light. J Opt Soc Am. 1958;48:777-84.
3. Kelly DH. Flicker fusion and harmonic analysis. J Opt Soc Am. 1961;51:917-8. PubMed PMID: 13752373.
4. Roufs JAJ. Dynamic properties of vision-I. Experimental relationships between flicker and flash thresholds. Vision Res. 1972;12:261-78.
5. Rovamo J, Raninen A, Donner K. The effects of temporal noise and retinal luminance on foveal flicker sensitivity. Vision Res. 1999;39:533-9.
6. de Lange H. Attenuation characteristics and phase-shift characteristics of the human fovea-cortex systems in relation to flicker-fusion phenomena. Delft: TU Delft; 1957.
7. Swanson WH, Ueno T, Smith VC, Pokorny J. Temporal modulation sensitivity and pulse-detection thresholds for chromatic and luminance perturbations. J Opt Soc Am A. 1987;4(10):1992-2005. PubMed PMID: 3430210.
8. von Wiegand TE, Graham N, Hood DC. Testing a computational model of light-adaptation dynamics. Vision Res. 1995;35(21):3037-51. PubMed PMID: 8533341.
9. Hecht S, Verrijp CD. The influence of intensity, color and retinal location on the fusion frequency of intermittent illumination. Proceedings of the National Academy of Sciences of the United States of America. 1933;19(5):522-35. PubMed PMID: 16587779; PubMed Central PMCID: PMC1086060.
10. Wald G. Human vision and the spectrum. Science. 1945;101(2635):653-8. doi: 10.1126/science.101.2635.653. PubMed PMID: 17777531.

11. Aguilar M, Stiles WS. Saturation of the rod mechanism of the retina at high levels of stimulation. Optica Acta. 1954;1:59-64.
12. Adelson EH. Saturation and adaptation in the rod system. Vision Res. 1982;22(10):1299-312. PubMed PMID: 7179751.
13. Sharpe LT, Fach CC, Nordby K, Stockman A. The incremental threshold of the rod visual system and Weber's law. Science. 1989;244(4902):354-6. PubMed PMID: 2711186.
14. Wyszecki G, Stiles WS. Color Science: concepts and methods, quantitative data and formulae. 2nd ed. New York: Wiley; 1982.
15. Kelly DH. Flicker. In: Jameson D, Hurvich LM, editors. Handbook of Sensory Physiology. VII/4. Berlin: Springer-Verlag; 1972. p. 273-302.
16. Watson AB. Temporal sensitivity. In: Boff K, Kaufman L, Thomas J, editors. Handbook of Perception and Human Performance. 1. New York: Wiley; 1986. p. 6-1-6-43.
17. Kelly DH. Diffusion model of linear flicker responses. J Opt Soc Am. 1969;59(12):1665-70. PubMed PMID: 5360496.
18. Duda RO, Hart PE. Use of the Hough transformation to detect lines and curves in pictures. Communications of the Association for Computing Machinery. 15(1):11-5.
19. Bode HW. Network analysis and feedback amplifier design. Princeton: van Nostrand; 1945.
20. Stiles WS. Mechanisms of Colour Vision. London: Academic Press; 1978.

Tables

Level	De Lange (1958)				Kelly (1961)		Roufs (1972)				Stockman *et al.* (2006)			
	V		L		DHK		HJM		RK		ML		MM	
	Slope	R^2	Slope	R^2	Slope	R^2	Slope	R^2	Slope	R^2	Slope	R^2	Slope	R^2
1	-12.61 ±0.35	0.997	-12.05 ±0.31	0.994	-13.40 ±0.83	0.986	-8.64 ±0.56	0.967	-6.64 ±0.38	0.981	-11.69 ±0.00	1.000	-9.17 ±0.55	0.989
2	-13.38 ±0.47	0.991	-12.53 ±0.53	0.989	-10.15 ±0.38	0.994	-8.58 ±0.47	0.965	-7.17 ±0.30	0.988	-12.50 ±0.71	0.994	-11.22 ±1.03	0.967
3	-14.33 ±0.58	0.990	-14.68 ±0.30	0.998	-16.40 ±1.05	0.976	-11.54 ±0.47	0.982	-10.03 ±0.35	0.990	-15.19 ±0.24	0.985	-15.15 ±1.13	0.962
4	-14.59 ±0.52	0.990	-13.17 ±0.45	0.993	-19.18 ±1.05	0.985	-12.84 ±0.78	0.975	-12.57 ±0.14	0.999	-17.79 ±1.61	0.961	-16.56 ±0.66	0.987
5	-14.37 ±0.16	0.999	-17.43 ±0.78	0.986	-27.25 ±1.84	0.978	-16.38 ±0.48	0.985	-14.02 ±0.53	0.990	-24.20 ±1.93	0.963	-22.78 ±0.76	0.988
6	-14.52 ±0.33	0.996	-17.04 ±0.99	0.983	-32.19 ±1.93	0.982	-19.54 ±0.77	0.986	-18.50 ±1.37	0.968	-28.95 ±1.34	0.989	-22.46 ±0.58	0.993
7	-21.61 ±0.74	0.991	-19.75 ±1.22	0.978			-23.00 ±0.84	0.953			-22.67 ±0.80	0.990	-21.53 ±0.68	0.989
8	-23.68 ±0.58	0.996					-23.03 ±0.69	0.989			-27.46 ±2.06	0.973	-24.61 ±0.72	0.990
9											-23.56 ±1.02	0.987		
10											-24.01 ±0.97	0.989		

Table **A**

Level	Rovamo *et al.* (1999)		Swanson *et al.* (1987)				Von Wiegand *et al.* (1995)			
	AR		WS		TU		VMC		TEW	
	Slope	R^2	Slope	R^2	Slope	R^2	Slope	R^2	Slope	R^2
1	--	--	-8.69 ±0.28	0.995	-13.34 ±2.00	0.917	-9.68 ±0.40	0.985	-9.38 ±0.88	0.942
2	--	--	-11.04 ±0.54	0.990	-10.78 ±0.23	0.999	-12.26 ±0.52	0.986	-10.84 ±0.69	0.976
3	--	--	-13.81 ±1.24	0.969	-10.11 ±0.80	0.981	-17.57 ±0.65	0.988	-17.88 ±0.93	0.976
4	-8.81 ±0.56	0.995	-27.08 ±3.89	0.942	-20.02 ±0.95	0.991	-21.95 ±0.91	0.990	-21.99 ±2.14	0.930
5	-14.97 ±1.68	0.963								
6	-16.20 ±0.95	0.993								
7	-17.74 ±1.42	0.987								
8	-27.67 ±1.56	0.994								
9										
10										

Table **A** (continued)

Best-fitting falling high frequency slopes in units of Hz per $\log_{10}$ unit and R^2 values for each fit. Levels are from low to high as listed in the keys of Figure 2-6. The high-frequency slope cannot be reliably estimated at the lowest three levels for Rovamo *et al.* (1999) because of limited data. For other details see text.

Level	De Lange (1958)				Kelly (1961)		Roufs (1972)				Stockman *et al.* (2006)			
	V		L		DHK		HJM		RK		ML		MM	
	fc	$\log_{10}(g)$	*fc*	$\log_{10}(g)$	*fc*	$\log_{10}(g)$	*fc*	$\log_{10}(g)$	*fc*	$\log_{10}(g)$	*Fc*	$\log_{10}(g)$	*fc*	$\log_{10}(g)$
1											5.79 ±0.62	7.43 ±0.17	5.65 ±0.31	7.71 ±0.08
2											7.97 ±0.49	7.66 ±0.10	8.14 ±0.39	7.80 ±0.07
3					11.45 ±0.36	8.98 ±0.05			7.93 ±0.27	7.43 ±0.06	9.96 ±0.53	7.68 ±0.08	10.55 ±0.44	7.84 ±0.06
4	8.29 ±0.28	8.48 ±0.06	8.87 ±0.30	8.68 ±0.05	18.91 ±0.68	9.07 ±0.05	7.07 ±0.24	7.76 ±0.06	9.83 ±0.32	7.38 ±0.05	12.83 ±0.64	7.75 ±0.07	12.87 ±0.53	7.65 ±0.05
5	9.87 ±0.33	8.31 ±0.05	12.18 ±0.43	8.49 ±0.05	26.42 ±1.04	8.82 ±0.06	11.59 ±0.40	7.97 ±0.04	13.57 ±0.52	7.39 ±0.06	17.69 ±0.92	7.66 ±0.07	17.48 ±0.73	7.54 ±0.05
6	11.44 ±0.39	8.24 ±0.05	14.21 ±0.53	8.33 ±0.05	33.36 ±1.38	8.19 ±0.06	15.19 ±0.57	7.82 ±0.04	20.26 ±0.90	6.99 ±0.06	20.76 ±1.24	7.44 ±0.09	16.48 ±0.67	6.74 ±0.05
7	15.29 ±0.56	7.85 ±0.04	17.07 ±0.61	8.25 ±0.04			22.18 ±0.97	7.58 ±0.05			21.14 ±0.98	6.72 ±0.06	16.39 ±0.67	6.07 ±0.05
8	17.04 ±0.64	7.02 ±0.04					17.89 ±0.61	6.59 ±0.04			18.51 ±1.00	5.84 ±0.08	16.39 ±0.66	5.44 ±0.05
9											19.24 ±0.93	5.34 ±0.06		
10											20.44 ±0.97	4.94 ±0.06		
k	0.81±0.01		0.83±0.01		0.90±0.01		0.80±0.01		0.78±0.01		0.78±0.01		0.84±0.02	

Table **B**

Level	Rovamo *et al.* (1999)		Swanson *et al.* (1987)				Von Wiegand *et al.* (1995)			
	AR		WS		TU		VMC		TEW	
	fc	$\log_{10}(g)$	*fc*	$\log_{10}(g)$	*fc*	$\log_{10}(g)$	*fc*	$\log_{10}(g)$	*fc*	$\log_{10}(g)$
1										
2			6.10 ±0.27	7.52 ±0.09	5.99 ±0.22	7.65 ±0.07	8.51 ±0.41	7.65 ±0.07	7.31 ±0.25	7.42 ±0.07
3			10.10 ±0.45	7.59 ±0.07	9.52 ±0.39	7.61 ±0.07	12.71 ±0.63	7.39 ±0.06	12.03 ±0.45	7.41 ±0.05
4	5.68 ±0.27	7.48 ±0.09	15.09 0.83	7.27 ±0.09	14.59 ±0.64	7.38 ±0.07	17.36 ±1.06	6.97 ±0.07	17.27 ±0.76	7.10 ±0.06
5	8.00 ±0.32	7.68 0.07								
6	9.95 ±0.44	7.68 ±0.07								
7	13.16 ±0.69	7.67 ±0.08								
8	17.31 ±1.14	7.63 ±0.10								
9										
10										
k	0.80±0.01		0.80±0.01		0.83±0.01		0.61±0.03		0.79±0.02	

Table **B** (continued)

Best-fitting variable corner frequencies (*fc*), logarithmic gains ($\log_{10}(g)$) and feedforward gain (*k*). Two LP-stages, which were fixed across observers, had corner frequencies of 30.92 ±2.23 Hz. Adjusted R^2 for simultaneous fit = 99.6% Levels are from low to high as listed in the keys of Figure 2-6. For details see text.
